# Targeting Tex10 Overcomes Oxaliplatin Resistance by Competitively Disrupting the Non‐Canonical BAF Complex in Colorectal Cancer

**DOI:** 10.1002/advs.76895

**Published:** 2026-07-29

**Authors:** Ping Xu, Jie Huang, Xiaojin Gao, Lin Deng, Yanheng Deng, Denglu Wang, Chunlei Yu, Yunxiang Zhang, Jingdong Li, Xiaocong Xiang

**Affiliations:** ^1^ Institute of Hepatobiliary‐Pancreatic‐Intestinal Diseases Affiliated Hospital of North Sichuan Medical College North Sichuan Medical College Nanchong China; ^2^ Sichuan Clinical Research Center for Digestive Diseases Affiliated Hospital of North Sichuan Medical College Nanchong China; ^3^ Department of Hepatobiliary Surgery The Fourth People's Hospital of Zigong Zigong China; ^4^ Institute of Materia Medica School of Pharmacy North Sichuan Medical College Nanchong China; ^5^ Institute of Basic Medicine School of Basic Medical Sciences and Forensic Medicine North Sichuan Medical College Nanchong China

**Keywords:** autophagy, BRD9 (Bromodomain‐containing protein 9), colorectal cancer, immune checkpoint blockade, oxaliplatin resistance, Tex10 (testis expressed protein 10)

## Abstract

Although a small subset of colorectal cancer (CRC) patients benefit from immunotherapy, oxaliplatin (OXA)‐based chemotherapy remains the first‐line treatment. However, the widespread development of OXA resistance poses a major clinical challenge, and the underlying molecular mechanisms remain incompletely understood. In this study, we found that Tex10 deficiency markedly enhances OXA sensitivity in CRC cells by inducing cytostatic autophagy in xenograft and patient‐derived organoid models. *Tex10* ^−/−^ mice exhibit markedly reduced tumor numbers and decreased p62 levels in inflammation‐induced CRC. Mechanistically, we determined that Tex10 competitively binds BRD9, thereby disrupting the BRD9‐BRG1 interaction within the non‐canonical BAF (ncBAF) complex, which suppresses AMBRA1 transcription and ULK1 ubiquitination to inhibit autophagy. Furthermore, in silico screening identified gemcitabine (GEM) as a potent Tex10 inhibitor. GEM directly binds to the N268 residue of Tex10, reducing Tex10 abundance and unleashing autophagy. This, in turn facilitates further lysosomal degradation of Tex10, forming a potent positive‐feedback loop to overcome OXA resistance. In addition, Tex10 deletion enhances the antitumor efficacy of immunotherapy by inhibiting lysosomal degradation of PD‐L1. Collectively, this study uncovers a previously unrecognized antitumor mechanism of GEM and identifies Tex10 as a promising therapeutic target for overcoming OXA resistance and potentiating immunotherapy in CRC.

## Graphical abstract

This study reveals that Tex10 drives oxaliplatin resistance in colorectal cancer by competitively binding BRD9 to disrupt the ncBAF complex, thereby suppressing AMBRA1 transcription and ULK1‐mediated autophagy. Gemcitabine is identified as a direct Tex10 inhibitor that restores autophagy and overcomes resistance. Targeting Tex10 also stabilizes PD‐L1 to enhance immunotherapy efficacy.

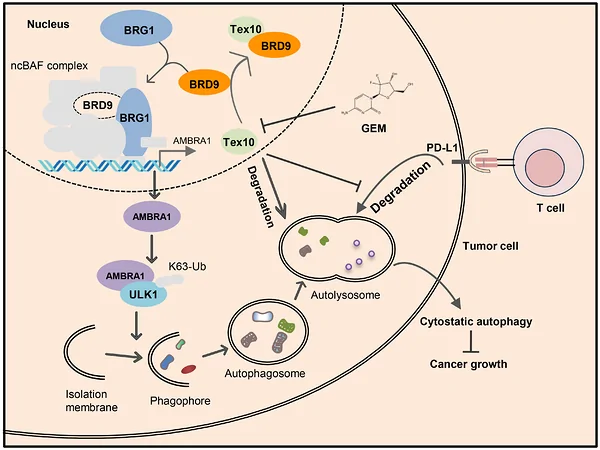

## 1 Introduction

Colorectal cancer (CRC) ranks as the third most common malignancy and the second leading cause of cancer‐related mortality worldwide [1]. Globally, an estimated 1.926 million new CRC cases and 904 000 CRC‐related deaths occurred in 2022 [2]. In China alone, approximately 517 000 new CRC cases, ranking second among all malignancies, and 240 000 CRC‐related deaths, ranking fourth overall, were reported in 2022 [3]. Treatment strategies for CRC include endoscopic or surgical resection, neoadjuvant radiotherapy, local ablation for metastatic disease, palliative chemotherapy, targeted therapy, and immunotherapy. However, 20%–25% of patients present with advanced or metastatic disease, precluding curative surgery. These individuals primarily receive systemic therapy comprising chemotherapy, targeted agents, and/or radiotherapy [4]. Approximately 85% of CRCs exhibit microsatellite stability (MSS) and respond poorly to immune checkpoint blockade (ICB), rendering most patients ineligible for immunotherapy [5, 6]. Oxaliplatin (OXA)‐based combination regimens remain the standard first‐line therapy for advanced CRC and are also used as adjuvant therapy after tumor resection [7, 8]. While initially effective, about 50% of patients eventually develop OXA resistance, leading to treatment failure and <10% 5 year survival [9]. Therefore, elucidating the molecular basis of OXA resistance is essential for improving therapeutic outcomes.

Tex10 (testis‐expressed protein 10) is a key transcriptional regulator implicated in the maintenance of self‐renewal and pluripotency in mouse embryonic stem cells [10, 11]. It also contributes to spermatogenesis and primordial germ cell differentiation [12] and plays critical roles in cell cycle progression and ribosome biogenesis [13]. Prior work suggests that Tex10 regulates tumor cell proliferation and migration through interactions with long non‐coding RNAs (lncRNAs) [14, 15]. Our previous work revealed that Tex10 binds STAT3 to promote hepatocellular carcinoma growth and metastasis [16]. Moreover, Tex10 activates NF‐κB signaling to enhance CRC cell proliferation [17]. Despite these findings, Tex10's functional role in cancer remains poorly defined, and its potential involvement in OXA resistance in CRC has not been investigated.

In this study, we demonstrate that Tex10 promotes OXA resistance in CRC, whereas Tex10 deficiency enhances autophagic flux and autophagosome formation. Mechanistically, Tex10 binds BRD9 and disrupts the BRD9–BRG1 interaction within the ncBAF complex, leading to suppression of AMBRA1 transcription and ULK1 ubiquitination, thereby inhibiting autophagy. Through in silico screening, we identified gemcitabine (GEM) as a potent small‐molecule inhibitor of Tex10 that binds directly to Tex10 and promotes its lysosome‐dependent degradation. GEM‐mediated inhibition of Tex10 significantly enhances OXA sensitivity both in vitro and in vivo. Additionally, Tex10 silencing augments anti‐tumor immunity by blocking lysosomal degradation of PD‐L1, and combining Tex10 inhibition with immunotherapy yields synergistic efficacy. Collectively, these findings establish Tex10 as a novel regulator of OXA resistance and highlight its potential as a druggable target for CRC therapy.

## 2 Materials and Methods

### 2.1 Cell Lines and Cell Culture

Human CRC cell lines HT29, HCT116, and HCT8 were obtained from the Cell Bank of the Chinese Academy of Sciences (Shanghai, China) and cultured in McCoy's 5A medium (Procell, PM150710) supplemented with 10% fetal bovine serum (FBS; Gibco, 10099–141C). RKO cells and OXA‐resistant human CRC cell lines HCT8‐R and HCT116‐R were purchased from MeisenCell (Zhejiang, China) and maintained in RPMI‐1640 medium (Procell, PM150110) supplemented with 10% FBS (Gibco, 10099–141C). Human HEK293T cells were obtained from the Cell Bank of the Chinese Academy of Sciences and cultured in DMEM (Gibco, C11995) containing 10% FBS. All cells were incubated at 37°C with 5% CO_2_ and confirmed to be free of mycoplasma contamination.

### 2.2 Human Samples

A total of 72 CRC tissue samples with corresponding clinical data were collected to analyze the correlation between Tex10 expression and OXA resistance. Among them, 43 patients received preoperative FOLFOX chemotherapy and 29 received postoperative FOLFOX chemotherapy. A tissue microarray containing 97 tumor tissues and 45 adjacent nontumor tissues from CRC patients was purchased from Outdo Biotech (Shanghai, China). All patients were treated at the Affiliated Hospital of North Sichuan Medical College (Nanchong, China). Written informed consent was obtained from all participants, and the study protocol was approved by the Ethics Committee of the Affiliated Hospital of North Sichuan Medical College [No. 2024ER653‐1].

### 2.3 Plasmids and Small Interfering RNAs

Plasmid constructs were generated using the Gibson Assembly cloning method. The cDNA sequences of Tex10, BRD9, and BRG1 were individually cloned into the pcDNA3.1‐FLAG, pcDNA3.1‐Myc, and pcDNA3.1‐HA vectors for transient expression. Additionally, BRD9 cDNA sequences containing domain deletions were cloned into the pcDNA3.1‐Myc vector to generate BRD9 deletion mutants. Site‐directed mutagenesis was used to construct the Tex10 mutants Tex10^N268A^ and Tex10^Y623A^, which were cloned into the pcDNA3.1‐FLAG vector. All constructs were verified by full‐length sequencing. Target sequences for shRNA and siRNA targeting AMBRA1, ULK1, and BRG1 are listed in Table S1. Primers used for qPCR and ChIP–qPCR analyses are provided in Table S2 and Table S3, respectively.

### 2.4 Cell Viability Assay

CRC cells growing well were counted using a hemocytometer, and cell density was adjusted to 3 × 10^4^ cells/mL with complete medium to prepare a homogeneous single‐cell suspension. The suspension was seeded into 96‐well plates (100 µL per well) and incubated overnight at 37°C in 5% CO_2_. After adhesion, cells were treated with OXA or GEM at concentrations ranging from 0–100 µM for 48 h, with three replicate wells per condition. After treatment, the medium was removed, and 100 µL of CCK‐8 reagent (1:10) was added to each well. Plates were incubated for 1 h, and absorbance at 450 nm was measured using a microplate reader (SpectraMax iD3, Molecular Devices).

For organoid viability assays, CRC organoids were cultured using the Colorectal Cancer Organoid Kit (serum‐free; Bio‐Techne, K2103‐CR). Approximately 100 spheroids per well were suspended in Matrigel diluted with culture medium and seeded in 96‐well plates. Organoid viability after 4 days of OXA or GEM treatment was measured using the CellTiter‐Glo 3D Cell Viability Assay Kit (Promega, G9683). Dose‐response curves were plotted and IC50 values calculated using GraphPad Prism 8.0 software to evaluate cell proliferation.

### 2.5 Apoptosis Analysis

CRC cells were seeded in 6‐well plates at a density of 2 × 10^5^ cells per well. When cells reached approximately 30%–40% confluence, they were treated with 30 µM oxaliplatin (OXA) for 24 or 48 h. Following treatment, cells were dissociated using EDTA‐free trypsin, collected into 15 mL conical centrifuge tubes, and centrifuged at 1000 ×g for 5 min at 4°C. The resulting cell pellets were resuspended in 200 µL of 1× Binding Buffer. According to the manufacturer's instructions, 5 µL of Annexin V‐APC (Vazyme, A214‐01) and 5 µL of Propidium Iodide (Vazyme, A214‐02) were added to each sample. After gentle mixing, cells were incubated for 20 min at room temperature in the dark. Immediately before analysis, 200 µL of additional Binding Buffer was added to adjust the final sample volume, and the cell suspension was passed through a 70 µm cell strainer. Apoptosis was quantified using a flow cytometer (NovoCyte Quanteon, Agilent).

### 2.6 Immunoblotting

Immunoblotting was conducted according to our previous study [18]. Cell lysates were prepared using RIPA lysis buffer (PC101, Epizyme, Shanghai, China) and analyzed by SDS‐PAGE. Proteins were transferred onto polyvinylidene difluoride (PVDF) membranes (Millipore, Bedford, MA, USA) and blocked with PBST containing 5% non‐fat dry milk. The primary antibodies used were as follows: anti‐FLAG (Sigma, F1804; 1:2000), anti‐FLAG (Immunoway, YM3809; 1:1000), anti‐HA (CST, 3724; 1:1000), anti‐Myc (CST, 2278; 1:1000,), anti‐Tex10 (Proteintech, 17372‐1‐AP; 1:1000,), anti‐LC3B (ZenBio, R381544; 1:1000), anti‐ULK1 (HuaAn, ET1704‐63; 1:2000), anti‐p62 (HuaAn, HA721171; 1:1000), anti‐β‐tubulin (ZenBio, R380628; 1:5000), anti‐GAPDH (HuaAn, ET1601‐4; 1:5000), Anti‐AMBRA1 (Santa Cruz,  sc‐398204; 1:250), Anti‐AMPK (HuaAn, ET1608‐40; 1:2000), anti‐phospho‐AMPK (Thr172) (CST, 2535; 1:1000,), Anti‐mTOR (HuaAn, ET1608‐5; 1:1000), anti‐phospho‐mTOR (Ser2448) (HuaAn, HA600094; 1:1000), anti‐BRG1 (HuaAn, ET1611‐85; 1:1000), anti‐BRD9 (Novus Biologicals, NBP2‐15614; 1:500). Membranes were incubated with primary antibodies overnight at 4°C, followed by incubation with HRP‐conjugated secondary antibodies (HuaAn, HA1001/HA1006; 1:10 000) for 1 h at room temperature. Signals were detected using the ECL Western Blotting Detection System (Merck Millipore, WBKLS0500) and visualized with a Fusion Solo imaging system (Vilber, France).

### 2.7 Mass Spectrometry Analysis of Tex10 Interaction

To identify proteins interacting with Tex10, HT29 cells were transfected with a plasmid encoding FLAG‐Tex10.48 h post‐transfection, cells were harvested and lysed in IP/Western lysis buffer (Beyotime, P0013) for 30 min. Lysates were subjected to immunoprecipitation using anti‐FLAG M2 magnetic beads (Sigma, M8823). The immunoprecipitated complexes were analyzed by liquid chromatography‐tandem mass spectrometry (LC‐MS/MS) at Jingjie Biotechnology (Hangzhou, China).

### 2.8 Immunofluorescence (IF)

CRC cells were cultured in 35 mm glass‐bottom dishes until reaching 70%–80% confluence and then fixed with 4% paraformaldehyde for 20 min. Cells were permeabilized with 0.1% Triton X‐100 for 10 min and blocked with 3% BSA for 1 h at room temperature. After three washes with PBS, cells were incubated overnight at 4°C with primary antibodies (anti‐ULK1 or anti‐LC3). Following three additional PBS washes, cells were incubated with Alexa Fluor 488 goat anti‐rabbit IgG (H + L) (Thermo Fisher Scientific, A11008; 1:200) for 1 h at room temperature. Nuclei were counterstained with DAPI for 10 min, and images were acquired using an Olympus FV3000 confocal microscope.

### 2.9 Immunoprecipitation

HEK293T cells were transfected with the indicated plasmids for 48 h. Cells were then lysed for 30 min in IP/Western lysis buffer (Beyotime, P0013) supplemented with a protease inhibitor cocktail. Lysates were clarified by centrifugation at 12 000 × g for 10 min at 4°C, and a 30 µL aliquot was retained as input. For FLAG‐IP, lysates were incubated with anti‐FLAG M2 magnetic beads (Sigma, M8823). For HA‐IP and Myc‐IP, lysates were incubated with Anti‐HA Magnetic Agarose (M180‐10, MBL) or Anti‐Myc Magnetic Agarose (MBL M047‐10), respectively. For anti‐BRD9‐IP, HCT116 cells were lysed as described above and incubated with anti‐BRD9 (Novus Biologicals; 1:100) or normal IgG (HuaAn EM2001‐04; 1:100) at 4°C for 16 h. Immunocomplexes were then incubated with Protein G Magnetic Beads (CST, 70024S) for 1.5 h. The bead‐bound complexes were washed three times with ice‐cold IP/Western lysis buffer and analyzed by Western blotting.

### 2.10 Detection of Autophagic Flux

Autophagic flux was detected according to our previous study [19, 20]. The RFP‐GFP‐LC3B lentivirus was purchased from Genechen (Shanghai, China). HCT116 and HT29 cells (4 × 10^5^ per well) were transduced with the lentivirus. After 72 h, cells were selected with puromycin (2.5 µg/mL) for one week to generate stable cell lines, which were subsequently infected with lentivirus shTex10. To assess autophagosome maturation, cells were seeded in 35 mm glass‐bottom dishes, treated with EBSS for 2 h or chloroquine (CQ; MCE, HY‐17589A) for 24 h, washed three times with PBS, and fixed with 4% paraformaldehyde for 20 min at room temperature. Images were captured using an Olympus FV3000 confocal microscope. For quantitative assessment of autophagic flux, cells treated with low‐serum DMEM or Bafilomycin A1 (Baf A1; MCE, HY‐100558) for 24 h were harvested, and RFP and GFP fluorescence intensities were analyzed using a FACSCalibur flow cytometer (BD Biosciences, Franklin Lakes, NJ). Changes in autophagic flux were determined by calculating RFP/GFP ratios from each quadrant of the flow cytometry plots.

### 2.11 Chromatin Immunoprecipitation (ChIP)

ChIP assays were performed according to the manufacturer's instructions (ChIP Kit, Millipore, Billerica, MA, USA). HT29 control and Tex10‐knockdown cells were cultured to 70%–80% confluence, fixed with 1% formaldehyde (CST, 12606) for 10 min at room temperature, and quenched with 125 mM glycine. Cells were washed twice with ice‐cold PBS containing 1 mM PMSF and 0.5 mM DTT (or protease inhibitor cocktail). Chromatin was sheared by sonication into uniform DNA fragments. Equal amounts of chromatin were incubated overnight at 4°C with rotation using either anti‐BRG1 antibody (Abcam, ab110641) or anti‐IgG (negative control; HuaAn, EM2001‐04) (2 µg per 1 × 10^7^ cells). Subsequently, 30 µL of Protein G Magnetic Beads were added to each sample and incubated for 2 h at 4°C with rotation. Protein/DNA complexes were collected by magnetic separation, eluted with elution buffer, and subjected to crosslink reversal. After DNA purification, PCR amplification was performed. All ChIP primer sequences are provided in Table S3.

### 2.12 Virtual Docking

Virtual docking was conducted by MedChemExpress Co., Ltd (Shanghai, China). Briefly: (1) the structural model of Tex10 (AlphaFold ID: AF‐Q9NXF1‐F1) was used for structural analysis and compound screening; (2) potential small‐molecule binding pockets were predicted using Schrödinger software, with Site 5 (key residues: GLU500, MET490, ILE53, etc.) prioritized for virtual screening based on the highest predicted binding scores; (3) compounds were sourced from MedChemExpress's FDA‐approved drug library (3102 compounds total); (4) 3D compound structures were generated in Schrödinger; (5) virtual screening was performed using the Virtual Screening Workflow module; and (6) binding affinities and molecular weights of potential Tex10‐interacting compounds were analyzed, yielding the top 200 small molecules with the highest docking scores.

### 2.13 Surface Plasmon Resonance

Binding affinities between GEM and Tex10 were determined using a Biacore T200 SPR system (GE Healthcare, USA) with a CM5 sensor chip. The CM5 chip was activated by injecting a freshly prepared 1:1 mixture of 400 mM EDC and 100 mM NHS at a flow rate of 10 µL/min for 420 s. Recombinant human Tex10 protein (Abnova, H00054881‐P01) was diluted to 4 µg/mL in immobilization buffer (10 mM sodium acetate, pH 3.5) and injected over the sample flow cell (Fc4) at 10 µL/min. The immobilization level achieved was approximately 3,350 response units (RU). The reference flow cell (Fc1) underwent no ligand immobilization. Surface deactivation was performed by injecting 1 M ethanolamine hydrochloride at 10 µL/min for 420 s. Gemcitabine monophosphate was serially diluted in running buffer (PBST containing 1% DMSO) to eight concentrations (200, 100, 50, 25, 12.5, 6.25, 3.125, and 0 µM) and injected across flow cells Fc1–Fc4 at a flow rate of 30 µL/min. The association and dissociation phases were recorded for 120 s and 200 s, respectively.

### 2.14 Cellular Thermal Shift Assay

HCT116 and HT29 cells were cultured in 10 cm dishes for 48 h. For the temperature‐dependent assay, cells were treated with DMSO or GEM (150 µM) at 37°C for 30 min, then equally divided into five aliquots and heated at the indicated temperatures for 3 min. Samples were cooled at room temperature for 3 min and lysed by three freeze‐thaw cycles in liquid nitrogen. For the dose‐dependent assay, cells were divided into five equal aliquots and treated with varying GEM concentrations (0–80 µM) for 30 min. Samples were then heated at 55°C for 3 min, cooled at room temperature for 3 min, and subjected to three freeze‐thaw cycles in liquid nitrogen to lyse the cells. All heated lysates were centrifuged at 13 000 × g for 20 min at 4°C, and the resulting supernatants were analyzed by SDS‐PAGE followed by immunoblotting.

### 2.15 Transmission Electron Microscopy

HT29 cells were infected with Tex10‐knockdown lentivirus (2 × 10^6^ cells/well). After 72 h, cells were selected with puromycin (2.5 µg/mL) to establish stable knockdown cell lines. Cells were then treated with GEM (5 µM) for 48 h, collected, and fixed with glutaraldehyde at room temperature for 2 h. The cell pellets were washed three times with PBS and embedded in 1% agarose. Samples were then fixed with 1% osmium tetroxide for 2 h in the dark, followed by three additional PBS washes. After dehydration through a graded ethanol series, samples were infiltrated with acetone‐812 resin and embedded. Ultrathin sections (60–80 nm) were stained with 2% uranyl acetate and 2.6% lead citrate in the dark. After drying, sections were examined and imaged using a transmission electron microscope.

### 2.16 Flow Cytometry Analysis

Tumor tissues were excised from mice and minced into 2–4 mm fragments. An enzyme cocktail was prepared according to the manufacturer's instructions (Tumor Dissociation Kit, mouse; Miltenyi Biotec, 130‐096‐730). Tissue fragments were transferred to gentleMACS C Tubes, and 3–5 mL of the enzyme cocktail was added. Single‐cell suspensions were generated using a pre‐programmed gentleMACS Dissociator protocol. The resulting suspensions were sequentially filtered through 70 and 30 µm MACS SmartStrainers to obtain isolated single cells for subsequent flow cytometry analysis. Cells were resuspended in 100 µL BD Stain Buffer and blocked with anti‐mouse CD16/CD32 antibody (TruStain FcX, BioLegend, 101319) for 10 min. After blocking, various fluorochrome‐conjugated antibodies were added, and surface staining was performed by incubating cells for 20 min at 4°C in the dark. Cells were washed twice with Stain Buffer (BD Pharmingen, 554656) and resuspended in 400 µL PBS for flow cytometry analysis. Fluorescence intensity was measured at designated wavelengths following the established gating strategy (Figure S1). Data were analyzed using FlowJo (v10.8.0).

### 2.17 Subcutaneous Tumor Model

All animal experiments were approved by the Ethics Committee of North Sichuan Medical College (NSMC Ethics Animal Review [2024]101). For in vivo subcutaneous tumor model experiments, 4–6‐week‐old male BALB/c nude mice or BALB/c immunocompetent mice were obtained from Beijing Vital River Laboratory Animal Technology Co., Ltd. and maintained under specific pathogen‐free (SPF) conditions. For OXA response evaluation, HT29‐scramble or Tex10‐knockdown cells were suspended at 3 × 10^6^ cells/100 µL PBS and injected subcutaneously into the left axilla of BALB/c nude mice. When tumor volumes reached approximately 60 mm^3^, mice were randomly divided into six groups (n = 5 per group): ① HT29‐scramble + normal saline group, ② HT29‐shTex10‐1 + normal saline group, ③ HT29‐shTex10‐2 + normal saline group, ④ HT29‐scramble + 3 mg/kg OXA group, ⑤ HT29‐ shTex10‐1 + 3 mg/kg OXA group, ⑥ HT29‐shTex10‐2 + 3 mg/kg OXA group. Drugs were administered via intraperitoneal injection every three days for two consecutive weeks.

For combination therapy, 3 × 10^6^ HT29 cells were suspended in 100 µL PBS and injected subcutaneously into the left axilla of BALB/c nude mice. When tumors reached approximately 60 mm^3^, mice were randomly assigned to four groups (n = 6 per group): ① control (normal saline); ② OXA group (3 mg/kg); ③ GEM group (40 mg/kg); ④ combination therapy group. Treatments were administered intraperitoneally every three days for three consecutive weeks.

For immunotherapy studies, 3 × 10^6^ CT26‐scramble or Tex10‐knockdown cells (in 100 µL PBS) were injected subcutaneously into the left axilla of 6‐week‐old immunocompetent BALB/c mice. When tumors reached 50 mm^3^, mice were randomly assigned to six groups (n = 7 per group): ① CT26‐scramble + control IgG group; ② CT26‐scramble + PD‐1 monotherapy group (100 µg/mouse, once every 5 days); ③ CT26‐scramble + PD‐L1 monotherapy group (100 µg/mouse, once every 5 days); ④ CT26‐shTex10 + control IgG group; ⑤ CT26‐shTex10 + PD‐1 combination therapy group; ⑥ CT26‐shTex10 + PD‐L1 combination therapy group. All drugs were administered via intraperitoneal injection every three days for a total of 12 days.

CT26 cells (3 × 10^6^ cells/100 µL PBS) were subcutaneously inoculated into the left axilla of 6‐week‐old immunocompetent male BALB/c mice. When the subcutaneous tumor volume reached approximately 50 mm^3^, the mice were randomly assigned to six groups (n = 5 per group): ① control (control IgG); ② PD‐1 group (100 µg/ mouse, once every 5 days); ③ PD‐L1 group (100 µg/ mouse, once every 5 days); ④ GEM group (20 mg/kg, once every 5 days); ⑤ GEM + PD‐1 combination therapy group; ⑥ GEM + PD‐L1 combination therapy group. All drugs were administered via intraperitoneal injection every 5 days for a total of 18 days. Tumor volumes were measured every 2 days using a caliper and calculated according to the formula: V = length × width^2^ × 0.5. At the end of the treatment period, mice were euthanized, and tumors, as well as major organs (heart, liver, spleen, lung, and kidney), were harvested and fixed in 10% neutral buffered formalin for further analysis.

Tex10^−/−^ mice were generated by crossing Tex10^flox/flox^ mice with Villin‐CreERT2 mice (The Jackson Laboratory). For the azoxymethane/dextran sulfate sodium (AOM/DSS)‐induced colitis‐associated carcinogenesis model, 2‐month‐old mice received an intraperitoneal injection of azoxymethane (AOM, 12.5 mg/kg). Seven days later, the mice were given 1.5% DSS in drinking water for 3 days, followed by 2 weeks of regular drinking water. This DSS cycle was repeated twice, and mice were sacrificed 12 weeks after the initial AOM injection.

### 2.18 Statistical Analysis

All statistical analyses were performed using GraphPad Prism 8.3. Comparisons between two groups were conducted using the Student's *t*‐test, while comparisons among multiple groups were performed using one‐way ANOVAs. All statistical tests were two‐sided, and a *P*‐value < 0.05 was considered statistically significant. All in vitro experiments were independently repeated at least three times, each including both biological and technical replicates.

## 3 Results

### 3.1 Tex10 Drives OXA Resistance in CRC

We first analyzed multiple public datasets to characterize Tex10 expression and its clinical relevance in CRC. Across both GEO and TCGA datasets, Tex10 mRNA levels were significantly upregulated in CRC tissues compared with normal controls (Figure 1A–D). Immunohistochemistry (IHC) on a tissue microarray containing 97 CRC samples confirmed elevated Tex10 protein expression (Figure 1E, F). Patients with high Tex10 expression exhibited significantly shorter overall survival (Figure 1G). To determine whether OXA treatment modulates Tex10 expression, we examined CRC cell lines exposed to OXA. Tex10 protein levels were increased in OXA‐treated HCT8 and HCT116 cells (Figure 1H), suggesting a role in the adaptive response to chemotherapy. To assess whether Tex10 overexpression contributes to OXA resistance, we compared parental and OXA‐resistant CRC sublines (HCT8‐R and HCT116‐R). The IC50 values for OXA in resistant sublines were at least threefold higher than those in parental cells (Figure 1I). Moreover, OXA‐induced apoptosis was significantly reduced in resistant cells (Figure 1J,K). Tex10 protein expression was markedly elevated in both HCT8‐R and HCT116‐R cells relative to their parental counterparts (Figure 1l). We further analyzed tumor tissues from 29 advanced CRC patients treated postoperatively with OXA‐based therapy and 43 stage II/III CRC patients receiving preoperative OXA‐based treatment. Based on imaging evaluations, patients were categorized as OXA‐sensitive or OXA‐resistant. Tex10 expression levels were significantly higher in the OXA‐resistant group (Figure 1M–O), and patients with high Tex10 expression exhibited shorter time to progression (TTP) (Figure 1P). Collectively, these results suggest that Tex10 overexpression contributes to OXA resistance in CRC.

**FIGURE 1:**
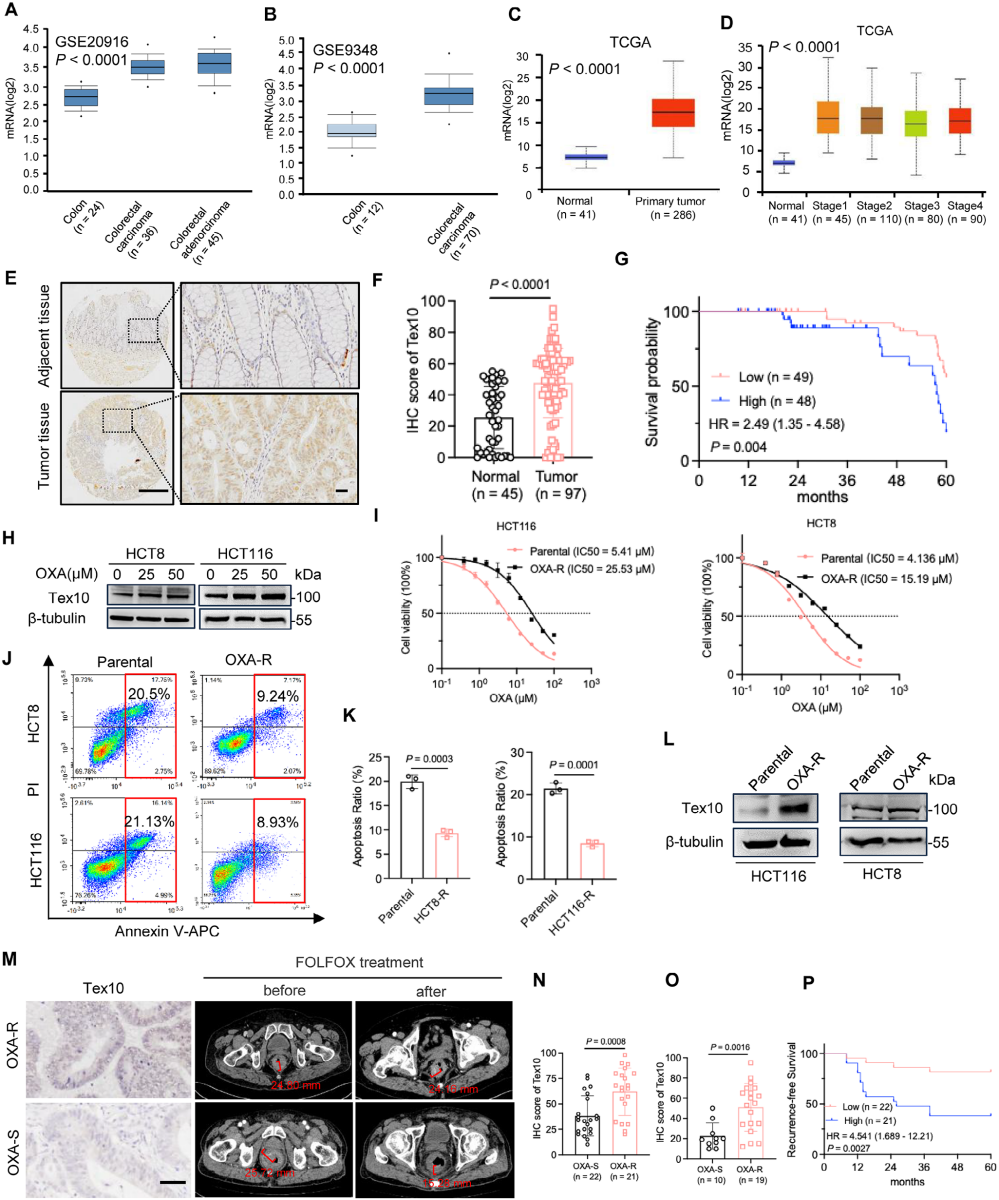
Tex10 is associated with OXA resistance and poor prognosis in CRC. (A–D) mRNA expression of Tex10 in human CRC tissues and normal tissues from the TCGA cohort. (E) Representative IHC images and statistical analyses of Tex10 expression in a tissue microarray. Scale bar: 200 µm (left) and 50 µm (right). (F) Examination of Tex10 expression in CRC (n = 97) and adjacent nontumor tissues in a microarray (n = 45). (G) Relationship between Tex10 expression and overall survival for tissue microarray. (H) The protein level of Tex10 in HCT‐8 and HCT116 cells treated with OXA (0, 25, and 50 µM) for 48 h. (I) The IC50 of OXA in HCT‐8 parental, HCT116 parental, HCT‐8‐R and HCT116‐R cells. (J, K) Apoptosis analysis of HCT‐8 parental, HCT116 parental, HCT‐8‐R and HCT116‐R cells treated with OXA (40 µM) using flow cytometry to detect Annexin V staining. (L) The protein‐level expression of Tex10 in OXA‐resistant HCT‐8 and HCT116 cells and the corresponding parental cells. (M) Representative IHC staining of Tex10 and computed tomography scans in OXA‐sensitive (OXA‐S) and OXA‐resistant (OXA‐R) patients. Scale bar: 50 µm. (N, O) Quantification of Tex10 staining in CRC tissues collected from OXA‐S and OXA‐R groups in stage II/III (N) and stage IV (O). (P) The correlation between Tex10 levels and Recurrence‐free Survival (RFS) for patients treated with OXA. HR, hazard ratio. Statistical significance in (F, N, O) was determined using two‐tailed paired t tests in GraphPad Prism. Statistical significance in (G, P) was determined with the log‐rank test. Error bars represent ± SD. Source data are provided as a Source Data file.

### 3.2 Tex10 Promotes OXA Resistance In Vitro and In Vivo

To capture both intrinsic and acquired OXA resistance mechanisms, we selected HCT116 (microsatelliteinstability‐high‌) and HT29 (microsatellitestable) alongside OXA‐resistant sublines HCT116‐R and HCT8‐R, allowing comprehensive evaluation of Tex10 function across diverse genetic contexts. To investigate the role of Tex10 in OXA resistance, we first examined the effects of Tex10 overexpression on the response of HCT116 and HT29 cells to OXA (Figure 2A). We found that Tex10 overexpression markedly reduced OXA‐induced apoptosis compared with vector controls (Figure 2B, C). We then assessed the effects of Tex10 knockdown on OXA sensitivity in HCT116, HT29, HCT116‐R, and HCT8‐R sublines. The knockdown efficiency of two independent shRNAs targeting Tex10 was confirmed by immunoblotting in these cell lines (Figure 2D and Figure S2A). Tex10 knockdown decreased the IC50 of OXA in all four cell lines (Figure 2E and Figure S2B). Furthermore, flow cytometric analysis using PI and Annexin V‐APC staining confirmed that Tex10 knockdown sensitized HCT116, HT29, HCT116‐R, and HCT8‐R cells to OXA‐induced apoptosis compared with control cells (Figure 2F and Figure S2C). To further explore the role of Tex10 in response to OXA treatment, we established CRC organoids. Following lentiviral knockdown of Tex10 and four days of OXA treatment, qPCR confirmed efficient Tex10 silencing (Figure 2G). Tex10 knockdown markedly enhanced OXA sensitivity (Figure 2H) and significantly reduced the OXA IC50 in CRC organoids (Figure 2I). Consistently, IHC staining of Ki67 showed that Tex10 depletion enhanced OXA efficacy, mirroring observations in CRC cells (Figure 2J,K).

**FIGURE 2:**
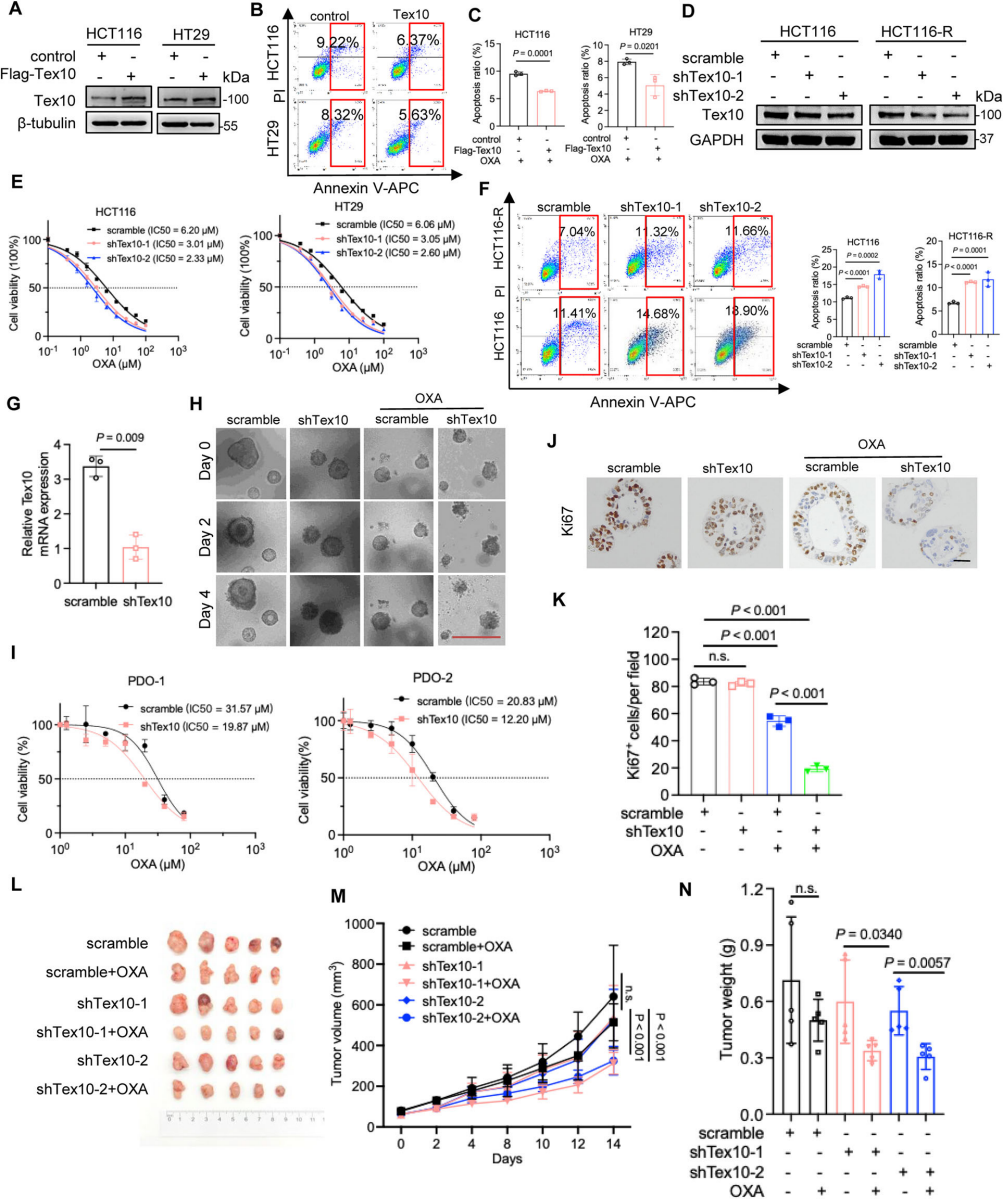
Tex10 promotes OXA resistance in vitro and in vivo. (A) Immunoblotting detection of the overexpression efficiency of Tex10 in HCT116 and HT29 cells. (B, C) The effect of Tex10‐overexpression on apoptosis following OXA treatment (10 µM) for 24 h as determined by flow cytometry. (D) Immunoblotting analysis of the knockdown efficiency of Tex10 in CRC cells. (E) Sensitivity to OXA in scramble (negative control shRNA) and shTex10 (Tex10‐targeted shRNA) cells. (F) The effect of Tex10 silencing on apoptosis following OXA treatment (30 µM, 48 h) as detected by flow cytometry. (G) qRT‐PCR analysis of Tex10 mRNA levels in Tex10‐silenced and scramble control organoids. (H) Representative bright‐field (BF) images showing organoid responses to OXA (30 µM). Scale bar: 200 µm. (I) The IC50 of OXA in CRC organoids upon Tex10 depletion. (J) IHC staining of organoids for Ki67. Scale bar: 10 µm. (K) Quantification of Ki67 staining in organoids. (L–N) BALB/c mice (n = 5) were inoculated orthotopically with 3 × 10^6^ HT29 cells transfected with control shRNA (scramble) or shRNA for Tex10 (shTex10‐1 and shTex10‐2) and treated with saline or OXA (3 mg/kg) for 2 weeks. HT29 tumor images (L), tumor volume (M), and tumor weight (N) were measured for 14 days. For A and D, n = 3 biologically independent samples. Data in C, E, G, and I are presented as mean ± SD and analyzed by two‐sided Student's *t*‐test. F, K, M and N are presented as mean ± SD and analyzed using one‐way ANOVAs. In L, n = 5 biologically independent samples. Source data are provided as a Source Data file.

To evaluate the role of Tex10 in OXA resistance in vivo, we established xenograft models in nude mice using microsatellite‐stable HT29 cells expressing shRNA targeting Tex10 or a non‐targeting control (scramble). The mice were then treated with either saline or OXA. Tex10 silencing significantly enhanced the sensitivity of HT29 tumors to OXA treatment, as evidenced by reduced tumor size and weight (Figure 2L–N). IHC analysis revealed decreased Ki67 expression and increased cl‐caspase 3 in tumors derived from Tex10 knockdown cells treated with OXA compared with controls (Figure S2D, E). Collectively, these results demonstrate that Tex10 promotes CRC cell proliferation and survival and contributes to OXA resistance both in vitro and in vivo.

### 3.3 Tex10 Deletion Promotes Growth‐Suppressive Autophagy

Autophagy plays a dual role in cancer, either promoting or inhibiting tumor cell survival and growth [21]. Given that targeting autophagy potentiates chemotherapy and facilitates tumor shrinkage [22, 23]. We investigated whether Tex10 promotes OXA resistance by regulating autophagy. The effect of Tex10 overexpression or knockdown on autophagy was determined based on the conversion of LC3‐I to LC3‐II. As shown in Figure 3A, Tex10 knockdown increased the LC3‐II/I ratio and promoted sequestosome 1 (SQSTM1/p62) degradation in HCT116 and HT29 cells. An elevated LC3‐II/I ratio reflects the accumulation of autophagic vesicles (AVs), which may result either from enhanced AV formation during early autophagy or impaired fusion of AVs with lysosomes at later stages [24]. To distinguish between these possibilities, we treated cells with Bafilomycin A1 (Baf A1), an inhibitor of late‐stage AV‐lysosome fusion that blocks microtubule‐associated protein 1 light chain 3 (MAP1LC3B/LC3) degradation in autolysosomes. Compared with control cells, Tex10 knockdown led to increased LC3‐II accumulation after Baf A1 treatment, indicating that Tex10 silencing enhances AV formation (Figure 3B). Low‐glucose treatment induces cellular starvation and increases autophagy initiation [25], and similar results were obtained under low‐glucose conditions (Figure S3A, B). Conversely, Tex10 overexpression suppressed these effects (Figure S3C). Transmission electron microscopy (TEM) further revealed increased numbers of both autophagic vesicles and autolysosomes in Tex10 knockdown cells (Figure 3C). Consistently, immunofluorescence staining for LC3B showed increased autophagic puncta in HCT116 and HT29 cells following Tex10 knockdown, confirming enhanced accumulation of autophagic vesicles (Figure S3D).

**FIGURE 3:**
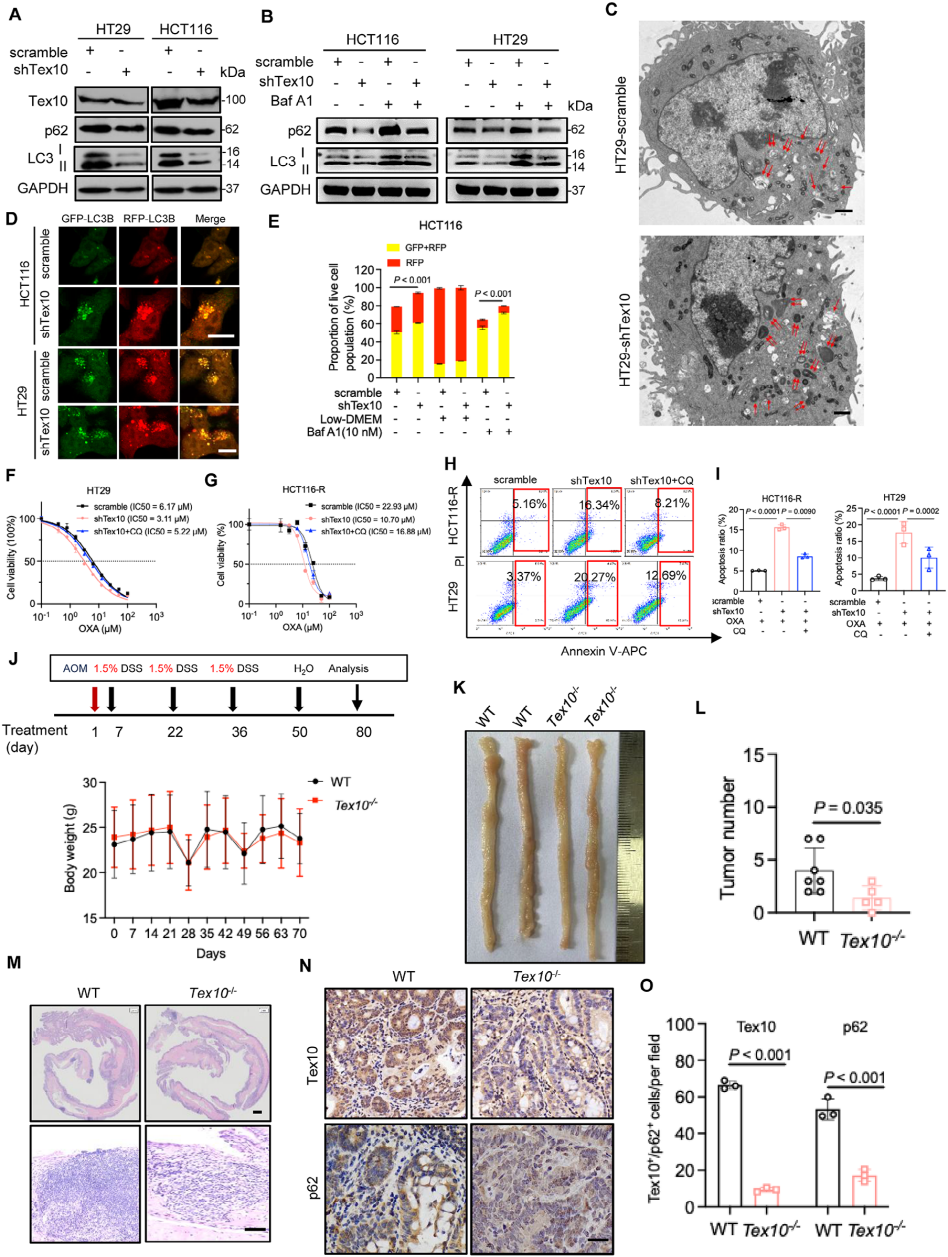
The depletion of Tex10 promotes OXA sensitivity by inducing cytostatic autophagy. (A) p62 and LC3B expression levels as measured by western blotting following the knockdown of Tex10. (B) Western blotting analysis of the indicated proteins in HCT116 and HT29 cells stably expressing shTex10 treated with or without 100 nM Baf A1 for 4 h. (C) Transmission electron micrographs of HT29 cells in which Tex10 was stably knocked down. Single arrows show the autophagosomes and double arrows show the autolysosomes. Scale bar: 10 µm. (D) Representative images of HCT116 or HT29 cells stably expressing shTex10 or a control vector were transfected with GFP‐mRFP‐LC3 to examine the expression of GFP and RFP by confocal microscopy. Scale bar: 10 µm. (E) CRC cells in (D) were cultured in complete or starvation medium treated with or without Baf A1 (10 nM) for 12 h. Quantitatively measured the expression of GFP and RFP by flow cytometry analysis. (F, G) CCK‐8 assay‐based confirmation of the effect of Tex10‐targeted knockdown on the OXA IC50 in CRC cells treated with or without 20 µM CQ for 24 h (F) or 48 h (G). (H, I) The effect of CQ on apoptosis (30 µM OXA) in Tex10‐knockdown CRC cells. (J) WT (n = 7) and *Tex10* ^−/−^ (n = 5) mice were injected with AOM on day 0 (2 months of age) and treated with 1.5% DSS for 3 days, followed by normal water for 2 weeks for 3 cycles. Body weight changes are recorded as indicated in J. (K, L) The number of tumors in the colon of WT and *Tex10* ^−/−^ mice. (M) Tumors in the colon as examined by H&E staining. Scale bar: 500 µm (upper) and 50 µm (bottom). (N, O) Representative images of Tex10 and p62 staining in the colon tumors of WT and *Tex10* ^−/−^ mice; quantification of Tex10/p62 is on the right (O). Scale bar: 50 µm. For A‐D and H, n = 3 biologically independent samples. Data E, F, G, and L are presented as mean ± SD and analyzed by one‐way ANOVA. J and L are presented as mean ± SD and analyzed using two‐sided Student's t tests. For M, N, and O, n = 3 biologically independent samples. Source data are provided as a Source Data file.

To assess autophagic flux, HCT116 and HT29 cells stably expressing RFP‐GFP‐LC3 were analyzed. In this system, autophagosomes appear yellow (RFP+GFP+), while mature autolysosomes appear red (RFP+GFP−) due to GFP quenching in the acidic lysosomal environment. Confocal microscopy showed that Tex10 knockdown increased the number of red puncta, indicating elevated autophagic flux and formation of mature autolysosomes (Figure 3D). Flow cytometric analysis of autophagic flux further confirmed that, after Baf A1 treatment, autophagosomes (RFP+GFP+) accumulated to a greater extent in Tex10‐silenced cells compared with controls (Figure 3E and Figure S3E). Together, these findings demonstrate that Tex10 suppresses autophagic flux in CRC cells.

To determine how Tex10‐regulated autophagy contributes to OXA resistance, we examined the effects of autophagy inhibition on OXA sensitivity. Treatment with chloroquine (CQ), an autophagy inhibitor, significantly increased the IC50 of OXA in Tex10‐silenced cells (Figure 3F), and similar results were obtained in HCT116‐R cells (Figure 3G). CQ treatment also abolished the pro‐apoptotic effects of Tex10 knockdown in HT29 and HCT116‐R cells treated with OXA (Figure 3H, I). Given that Tex10 depletion inhibits CRC proliferation [17], these findings indicate that Tex10 silencing modulates OXA resistance by inducing growth‐inhibitory autophagy. To further validate these observations in vivo, we generated an intestinal epithelial cell‐specific Tex10 knockout mouse model. Knockout efficiency was confirmed by measuring Tex10 protein expression via IHC (Figure S4A,B). *Tex10^−/−^* mice showed no changes in body weight or structural abnormalities compared with wild‐type (WT) littermate controls (Figure S4C,D). We then induced CRC by AOM/DSS to examine Tex10‐mediated autophagy (Figure 3J). Tex10 knockout reduced the number of tumors without affecting body weight (Figure 3K–M). Immunohistochemical analysis revealed decreased expression of p62 in Tex10‐knockout tumor tissues, consistent with our in vitro data (Figure 3N, O). Collectively, these results demonstrate that Tex10 deletion inhibits CRC progression by inducing growth‐suppressive autophagy while priming cells for enhanced OXA‐induced cell death.

### 3.4 Tex10 Suppresses ULK1 Protein Stability

To determine whether Tex10‐regulated autophagy involves the AMPK/mTOR signaling pathway, we analyzed the phosphorylation levels of mTOR and AMPK. In Tex10‐knockdown CRC cells (HCT116 and HT29), phosphorylated AMPK (p‐AMPK) expression was downregulated, while phosphorylated mTOR (p‐mTOR) levels remained largely unchanged (Figure 4A). Given that AMPK positively regulates autophagy initiation [26], the observed reduction in p‑AMPK contradicts the expected response upon Tex10 silencing‑induced autophagy, suggesting that Tex10 may regulate autophagy through alternative mechanisms. ULK1 is the key serine/threonine kinase essential for autophagy initiation. Tex10 silencing did not alter ULK1 mRNA levels but significantly increased ULK1 protein abundance, whereas Beclin‐1 levels remained unchanged (Figure 4B,C), indicating post‑translational regulation of ULK1 by Tex10. ULK1 is modulated through various post‐translational modifications (PTMs), including phosphorylation, acetylation, and ubiquitination [27, 28, 29]. Notably, K63‐linked ubiquitination promotes ULK1 kinase activity and stability, whereas K48‐linked ubiquitination leads to its degradation [30].

**FIGURE 4:**
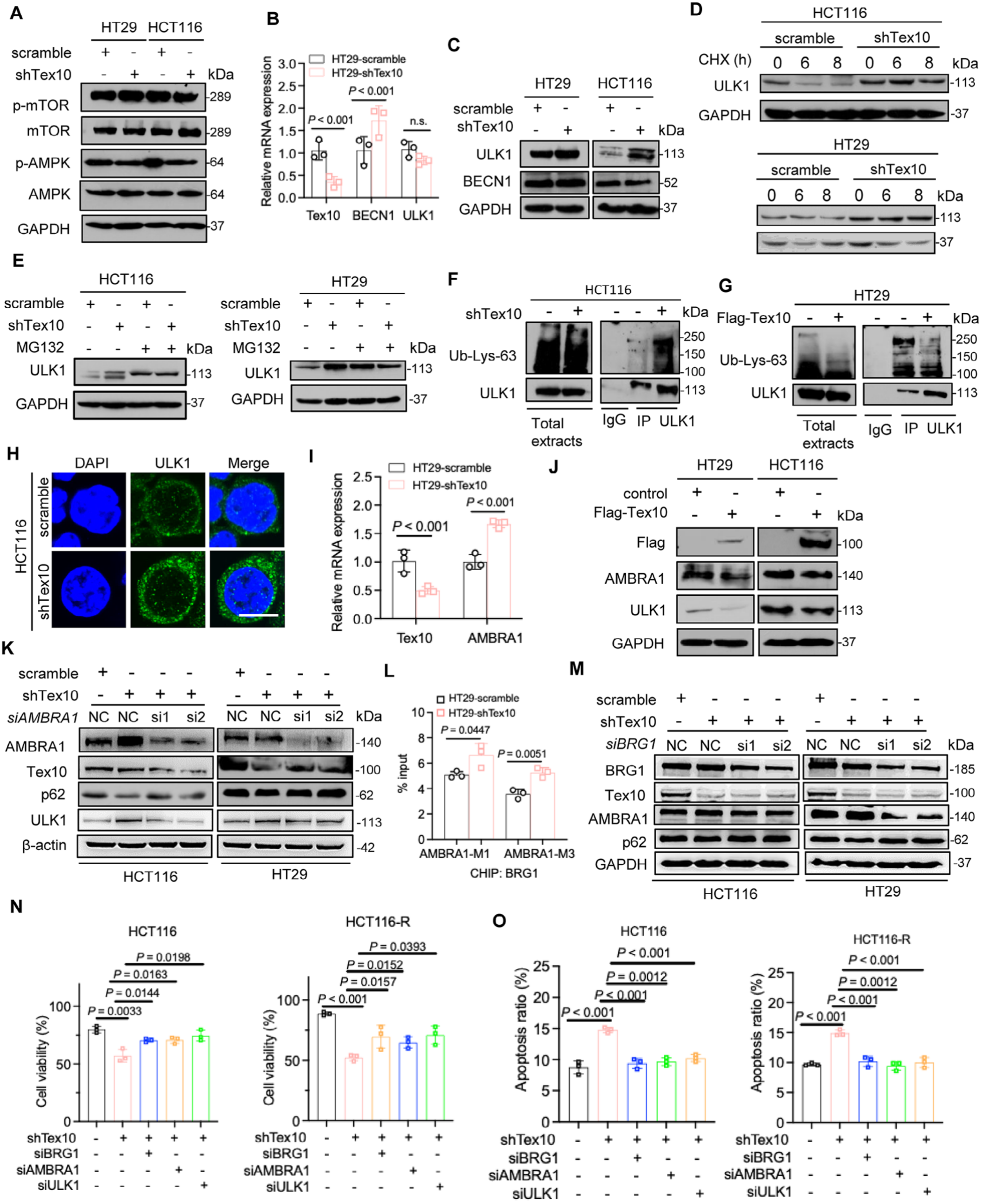
BRG1/AMBRA1 is required for Tex10‐mediated ULK1 ubiquitylation and autophagy inhibition. (A) mTOR, p‐mTOR, AMPK, and p‐AMPK expression levels were measured by western blotting following the knockdown of Tex10. (B, C) qRT‐PCR (B) and Western blotting (C) were used to detect the BECN1 and ULK1 mRNA and protein expression in Tex10‐silenced and scramble control cells. (D) Western blotting analysis of ULK1 expression in Tex10‐silenced HCT116 or HT29 cells treated with or without 35 µM cycloheximide for the indicated time. (E) Western blotting analysis of ULK1 expression in Tex10‐silenced CRC cells treated with or without 20 µM MG132 for 6 h. (F, G) Immunoprecipitation assay performed using anti‐ULK1, followed by western blotting with the indicated antibodies in Tex10‐silenced HCT116 (F) or Tex10‐overexpressing HT29 (G) cells. (H) Representative confocal images of ULK1 in HCT116 cells; the nucleus was stained with DAPI. Scale bar: 10 µm. (I) qRT‐PCR detection of AMBRA1 mRNA levels in Tex10‐silenced and scramble control cells. (J) Western blotting analysis of the expression of AMBRA1 and ULK1 in CRC cells overexpressing Tex10 and vector control cells. (K) HCT116 and HT29cells stably expressing shTex10 or control vector were transfected with siScramble or siAMBRA1 for 48 h. Protein levels of AMBRA1, Tex10, p62, and ULK1 were then detected by immunoblotting. (L) Chromatin immunoprecipitation (ChIP) analysis performed with anti‐BRG1 followed by detection of the AMBRA1 promoter through qPT‐PCR in HT29 cells stably expressing shTex10 or control vector. (M) HCT116 and HT29 cells stably expressing shTex10 or a control vector were transfected with siScramble or siBRG1 for 48 h. Protein levels of BRG1, AMBRA1, Tex10, and p62 were detected by immunoblotting. (N) The effect of BRG1/AMBRA1/ULK1 knockdown on cell proliferation of HCT116 (30 µM OXA)) and HCT116‐R (60 µM OXA) cells. (O) The effect of BRG1/AMBRA1/ULK1 knockdown on apoptosis of HCT116 (20 µM OXA) and HCT116‐R (60 µM OXA) cells. For A‐O, n = 3 biologically independent samples. B, I, L, N and O are presented as mean ± SD and were analyzed using two‐sided Student's t tests. Source data are provided as a Source Data file.

To assess ULK1 protein stability, cells were treated with the protein synthesis inhibitor cycloheximide (CHX). ULK1 degradation was markedly slowed following Tex10 silencing (Figure 4D), and treatment with the proteasome inhibitor MG132 reversed the Tex10 knockdown‐induced increase in ULK1 levels (Figure 4E), indicating that Tex10 suppresses ULK1 protein stability via the proteasomal ubiquitination pathway. Co‐immunoprecipitation (Co‐IP) assays further showed that K63‐linked ubiquitination of ULK1 increased upon Tex10 knockdown but decreased with Tex10 overexpression (Figure 4F,G). Consistently, immunofluorescence staining revealed increased ULK1 ubiquitination puncta in Tex10‐silenced cells (Figure 4H). Collectively, these results demonstrate that Tex10 inhibits the K63‐linked ubiquitination of ULK1, thereby destabilizing ULK1 protein and suppressing autophagy in CRC cells.

### 3.5 Tex10 Inhibits ULK1 Stability by Disrupting the BRD9‐Containing ncBAF Complex

Autophagy and Beclin 1 regulator 1 (AMBRA1) is a positive regulator of autophagy that promotes K63‐linked ubiquitination of ULK1 by binding to the E3 ubiquitin ligase TRAF6 [31]. Tex10 silencing upregulated AMBRA1 mRNA, whereas Tex10 overexpression downregulated both AMBRA1 and ULK1 protein levels, suggesting that Tex10 suppresses AMBRA1 transcription (Figure 4I,J). To determine whether Tex10‐mediated changes in ULK1 depend on AMBRA1, we co‐silenced AMBRA1 and Tex10. Compared with Tex10 knockdown alone, dual silencing reduced ULK1 expression to baseline levels and restored p62 expression (Figure 4K), indicating that AMBRA1 is involved in Tex10‐regulated ULK1 stability. The ncBAF complex is a chromatin‐remodeling complex, and BRG1, a central ATPase in this complex, regulates autophagy in mouse intestinal epithelial cells [32]. We therefore hypothesized that Tex10 participates in the BRG1‐mediated transcriptional regulation of AMBRA1. ChIP‐qPCR analysis revealed increased enrichment of BRG1 at the AMBRA1 promoter region after Tex10 silencing (Figure 4L), and dual silencing of BRG1 and Tex10 reduced AMBRA1 expression and restored p62 levels (Figure 4M), demonstrating that Tex10 regulates AMBRA1 transcription through BRG1. Together, these results indicate that Tex10 knockdown enhances BRG1‐mediated transcription of AMBRA1, thereby promoting ULK1 K63‐linked ubiquitination stability. To further investigate the functional relevance of the BRG1/AMBRA1/ULK1 axis in Tex10‐mediated OXA resistance, we simultaneously silenced Tex10 together with AMBRA1, BRG1, or ULK1, and examined CRC cell proliferation and apoptosis in the presence of OXA. Compared with control siRNA‐transfected cells, knockdown of AMBRA1, BRG1, or ULK1 in Tex10‐silenced cells significantly reversed OXA‐induced growth suppression and apoptosis (Figure 4N,O and Figure S5). Taken together, our data demonstrate that BRG1/AMBRA1/ULK1‐mediated growth‐inhibitory autophagy is required for Tex10‐mediated OXA resistance.

To further elucidate how Tex10 regulates the BRG1/AMBRA1 axis, we performed Co‐IP followed by mass spectrometry analysis and identified Bromodomain‐containing protein 9 (BRD9) as a Tex10‐interacting protein (Figure 5A). The interaction between Tex10 and BRD9 was confirmed by Co‐IP in HEK293T cells co‐expressing both proteins (Figure 5B), and endogenous interaction was further verified in HT29 cells (Figure 5C). To map the BRD9 domain responsible for binding Tex10, a series of BRD9 truncation mutants were generated (Figure 5D). Co‐expression of these mutants with Tex10 in HEK293T cells revealed that the DUF domain of BRD9 is essential for the interaction (Figure 5E). Given that BRD9 interacts with BRG1 to form part of the ncBAF complex, a smaller variant of the SWI/SNF chromatin remodeling complex [33, 34]. We hypothesized that Tex10 competitively disrupts the BRD9‐BRG association. Consistent with this hypothesis, co‐expression of Tex10, BRD9, and BRG1 resulted in a diminished interaction between BRG1 and BRD9 in the presence of Tex10, thereby confirming the competitive disruption (Figure 5F). Truncation analysis further revealed that both the N‐terminal and DUF domains of BRD9 are required for BRG1 binding (Figure 5G), consistent with a model in which Tex10 binds the DUF domain to competitively interfere with BRG1. Notably, when autophagy was induced by EBSS‐mediated nutrient starvation, the Tex10‐BRD9 interaction was disrupted (Figure 5H,I), indicating that the inhibitory effect of Tex10 on the pro‐autophagic function of the ncBAF complex is relieved under starvation conditions. Collectively, these results demonstrate that Tex10 competitively binds the DUF domain of BRD9, disrupting the BRD9‐BRG1 interaction within the ncBAF complex. This disruption suppresses AMBRA1 transcription, reduces ULK1 ubiquitination, and ultimately inhibits autophagy in CRC cells.

**FIGURE 5:**
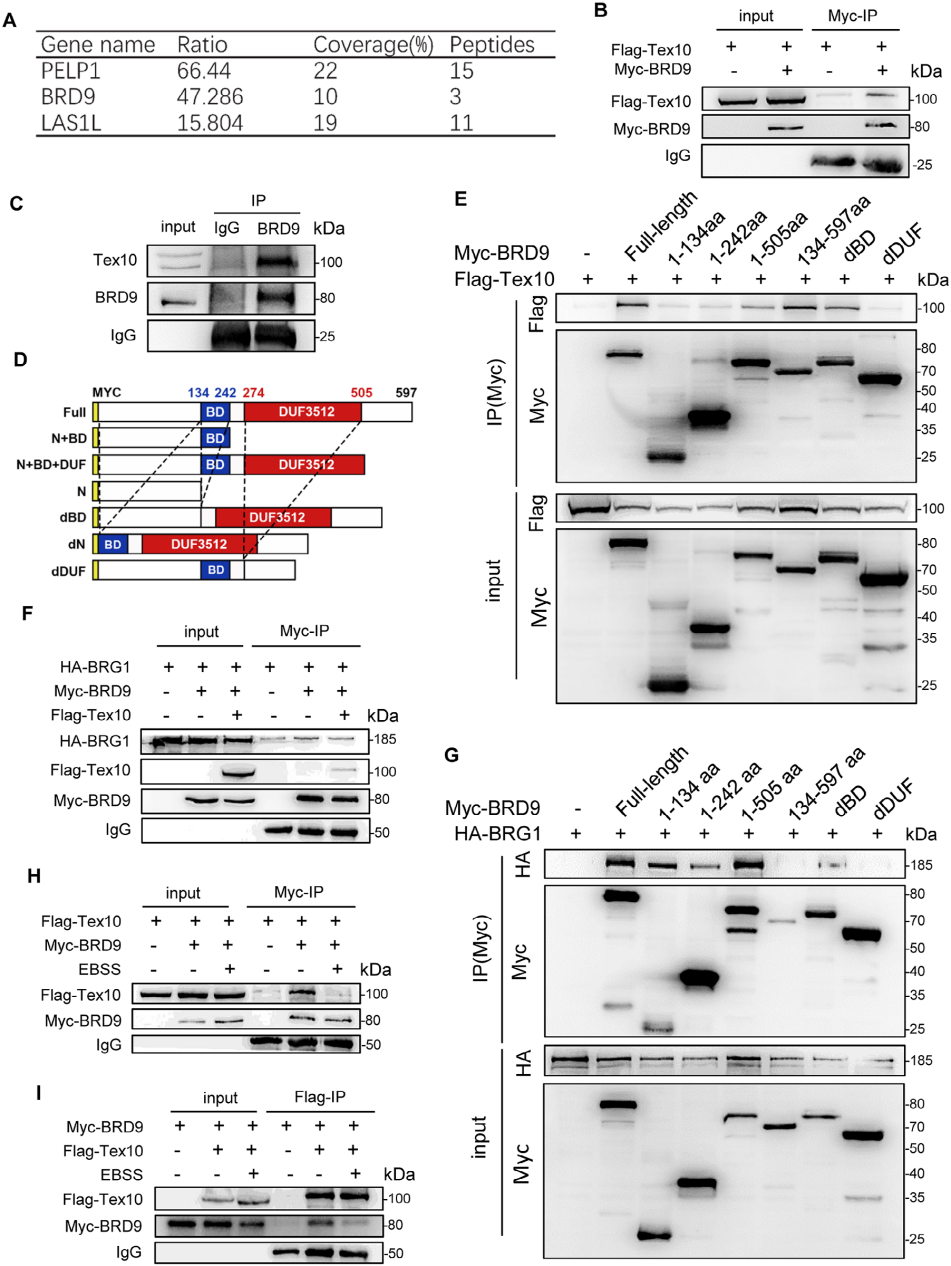
Tex10 interacts with BRD9 to disrupt the BRG1‐BRD9 complex. (A) Protein mass spectrometry data for Tex10 immunoprecipitates from HT29 cells. (B) Co‐IP assays conducted to verify the interaction between exogenous Tex10 and BRD9 in HEK293T cells. (C) Co‐IP assays conducted to investigate the interaction between endogenous Tex10 and BRD9 in HCT116 cells. (D) Schematic diagram of the BRD9‐Myc structural domain deletion construct. (E) Co‐IP assay conducted to detect the interaction between Tex10 and BRD9 structural domains in HEK293T cells. (F) HEK293T cells were co‐transfected with vectors encoding BRG1 ‐HA, BRD9‐Myc, and Tex10‐FLAG. Protein extracts were immunoprecipitated using anti‐Myc. (G) Co‐IP assay conducted to detect the interaction between BRG1 and BRD9 structural domains in HEK293T cells. (H, I) HEK293T cells cotransfected with the indicated plasmids for 48 h were incubated with or without EBSS for another 2 h, and then subjected to IP using anti‐Myc (H) or anti‐Flag (I) followed by Western blotting. For B, C, E, and F‐G, n = 3 biologically independent samples.

### 3.6 GEM Directly Binds to Tex10 and Promotes its Lysosome‐Dependent Degradation

Given the critical role of Tex10 in promoting OXA resistance in CRC cells, we performed virtual screening to identify small‐molecule inhibitors of Tex10. An FDA‐approved compound library was used for molecular docking against the Tex10 three‐dimensional structure predicted by AlphaFold (Figure 6A). Sixteen top‐ranked candidate molecules were selected for preliminary screening of antitumor activity in OXA‐resistant cell lines. Among these, eight compounds exhibited notable antitumor activity (Figure 6B). Based on their potential to inhibit Tex10 and p62 expression, these eight hit molecules (40 µM) were used to treat CRC cells for 24 h. Among them, only gemcitabine monophosphate significantly and simultaneously downregulated both Tex10 and p62 (Figure 6B, C). Further treatment of CRC cells with increasing concentrations of GEM for 48 h confirmed dose‐dependent downregulation of Tex10 and p62 proteins (Figure 6D). To verify direct GEM‐Tex10 binding, we performed cellular thermal shift assays (CETSA). Treating CRC cell lysates with GEM at various temperatures revealed that GEM enhanced the thermal stability of Tex10 (Figure 6E). Treatment of intact CRC cells with different GEM concentrations produced a dose‐dependent increase in Tex10 thermal stability, indicating intracellular GEM‐Tex10 binding (Figure 6F). Surface plasmon resonance (SPR) analysis confirmed direct binding between GEM and Tex10, with a dissociation constant (KD) of 3.51 µM (Figure 6G). Together, these findings demonstrate that GEM directly binds to Tex10 both in vitro and in cells.

**FIGURE 6:**
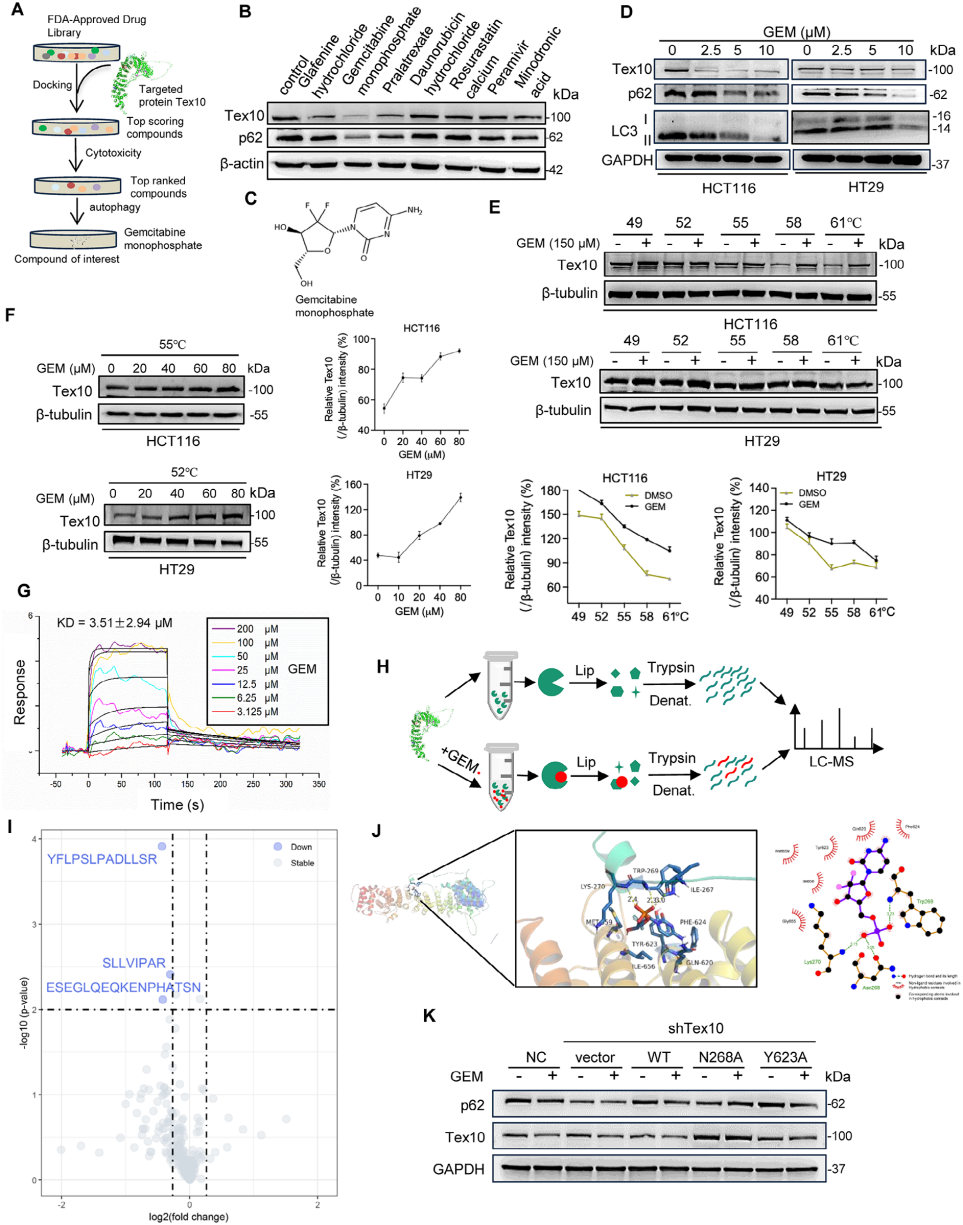
GEM directly targets Tex10. (A) Overview of the approach to the in silico screening of small‐molecule inhibitors of Tex10. (B) Western blotting analysis of Tex10 and p62 in HCT116‐R cells treated with indicated compounds for 24 h. (C) Structure of gemcitabine monophosphate. (D) Immunoblotting analysis of Tex10, p62, and LC3B expression in CRC cells treated with indicated concentrations of GEM for 24 h. (E) CETSA analysis of intracellular binding between GEM and Tex10. Protein levels were investigated at different temperatures under GEM (150 µM) treatment in HCT116 and HT29 cells for 30 min. Quantification of Tex10 in the CETSA assays is shown on the right. (F) Protein levels were investigated at 55°C or 52°C under the indicated concentrations of GEM in HCT116 and HT29 cells for 30 min. Quantification of the Tex10 intensity is shown on the right. (G) SPR analysis of GEM binding to the recombinant Tex10 protein. (H) Flowchart of the LiP‐MS experiment. (I) Quantitative analysis of differential peptide segments in a volcano plot. P < 0.01 and a difference multiple of more than 1.2 times were used as the screening criteria for differential peptide segments. (J) Molecular docking of GEM in the allosteric site of human Tex10. (K) Immunoblotting analysis of Tex10 and p62 levels in HT29 and Tex10 shRNA HT29 cells treated with GEM (5 μм) for 24 h. Prior to GEM treatment, HT29 cells stably expressing shTex10 were transfected with control vector, WT, and the mutant Tex10‐expressing plasmids pcDNA3.1‐Tex10^N268A^and pcDNA3.1‐Tex10^Y623A^, followed by quantification via densitometric analysis (n = 3). HT29 cells were used as a negative control (NC). For B, D, E, F, G, I, and K, n = 3 biologically independent samples. Data represent the mean ± SD of three biological replicates. Source data are provided as a Source data file.

To identify the Tex10 regions responsible for GEM binding, limited proteolysis‐mass spectrometry (LiP‐MS) was employed. The experimental workflow is outlined in Figure 6H. Recombinant human Tex10 (2 µg/100 µL) was incubated with either DMSO or 20 µM GEM for 10 min, followed by limited proteolysis with proteinase K for 5 min. After quenching enzyme activity, samples were completely digested with trypsin and analyzed by quantitative mass spectrometry. LiP‐MS identified three peptide regions (residues 247–263, 623–635, and 739–746) showing significant conformational changes upon GEM treatment (Figure 6I and Figure S6). Molecular docking of GEM with the Tex10 monomeric structure (UniProt ID: Q9NXF1) using AutoDock Vina predicted a binding energy of −6.0 kcal/mol (Figure 6J). GEM potentially forms hydrogen bonds with residues N268, S269, and D270 (C‐terminal region of peptide 247–263) and hydrophobic interactions with residues L620, Y623, and F624 (within peptide 623–635). To validate these predictions, mutant constructs Tex10^N268A^and Tex10^Y623A^ were generated and transfected into stable Tex10‐knockdown CRC cells. Mutation of Y623 to alanine (Y623A) did not affect GEM‐mediated autophagy function (downregulation of p62), whereas mutation of N268 to alanine (N268A) abolished GEM's effects (Figure 6K). These findings demonstrate that GEM specifically binds to the N268 residue of Tex10.

To assess the effect of GEM on autophagy in CRC cells, TEM was employed to examine changes in autophagosomes and autolysosomes following GEM treatment of HT29 cells for 48 h. The results showed that GEM promoted autophagic vesicle maturation in CRC cells (Figure 7A). Compared to treatment with Baf A1 alone, the combination of GEM and Baf A1 led to a further accumulation of LC3‐II, suggesting that GEM enhances autophagic vesicle formation (Figure 7B), phenocopying Tex10 silencing. To further determine whether GEM‐induced autophagy depends on Tex10, GEM was added to CRC cells stably silenced for Tex10. In these Tex10‐knockdown cells, GEM failed to further reduce Tex10 or p62 protein levels, indicating that the autophagy‐regulating effect of GEM in CRC cells is Tex10‐dependent (Figure 7C). Additionally, validation in HEK293T cells revealed that GEM disrupted the Tex10‐BRD9 interaction in a dose‐dependent manner (Figure 7D). To explore the mechanism by which GEM inhibits Tex10 function, qPCR analysis showed that GEM treatment upregulated Tex10 mRNA levels, a finding inconsistent with the observed decrease in Tex10 protein levels (Figure 7E). This suggests that GEM does not regulate Tex10 at the transcriptional level but rather affects its protein stability post‐translationally. Treatment with the protein synthesis inhibitor CHX, combined with GEM, confirmed that GEM reduces Tex10 protein stability (Figure 7F). To determine whether GEM‐mediated suppression of Tex10 protein stability occurs via the proteasomal or lysosomal degradation pathway, CRC cells were treated with GEM in combination with MG132, Baf A1, or CQ. Baf A1, but not MG132, rescued GEM‐induced Tex10 degradation, indicating that GEM promotes the lysosome‐dependent autophagic degradation of Tex10 (Figure 7G, H). Importantly, the Tex10‐N268A mutant completely abrogated GEM‐induced Tex10 reduction (Figure 6K). This result excludes off‐target DNA synthesis inhibition or general cytotoxicity and confirms that autophagic degradation is specifically triggered by direct GEM–Tex10 binding. Collectively, these results demonstrate that GEM directly binds to Tex10 at residue N268 and promotes its lysosome‐dependent autophagic degradation, thereby regulating autophagy in a Tex10‐dependent manner in CRC cells.

**FIGURE 7:**
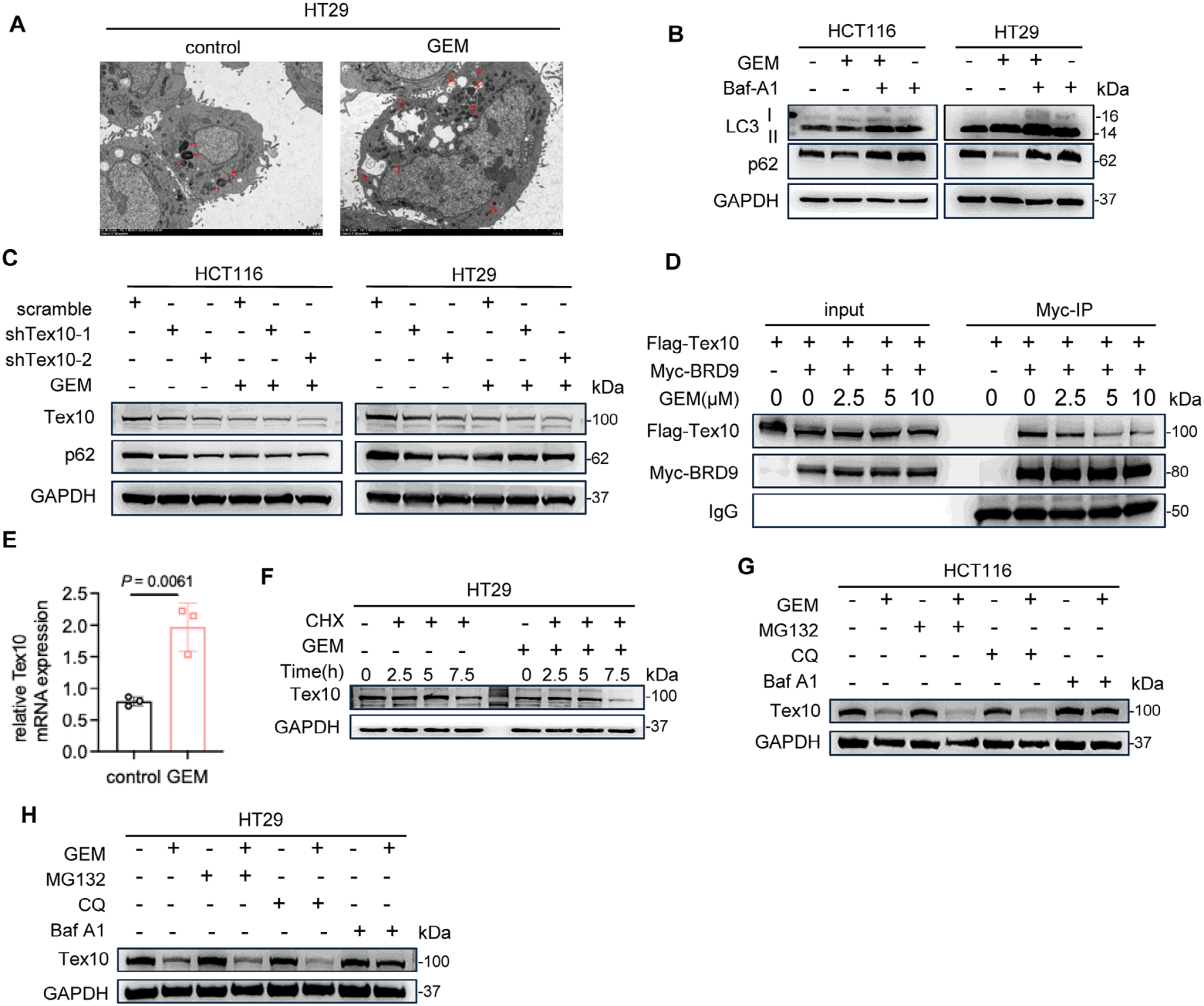
GEM induces the lysosome‐dependent degradation of Tex10. (A) TEM analysis of autophagosomes and autolysosomes in HT29 cells treated with or without 5 µM GEM. (B) Immunoblotting analysis of LC3B and p62 expression in HCT116 or HT29 cells treated with or without 5 µM GEM for 44 h, followed by the addition of 100 nM Baf A1 for an additional 4 h. (C) HCT116 or HT29 cells stably expressing shTex10 or a control vector were treated with or without 5 µM GEM for 24 h. Protein levels of Tex10 and p62 were then detected by immunoblotting. (D) HEK293T cells were co‐transfected with vectors encoding BRD9‐Myc and Tex10‐Flag for 48 h, followed by treatment with different concentrations of GEM for 24 h. Protein extracts were immunoprecipitated using anti‐Myc. (E) qRT‐PCR detection of the level of Tex10 expression in HCT116 cells treated with or without 2.5 µM GEM for 48 h. (F) Immunoblotting detection of Tex10 abundance in HT29 cells treated with DMSO or GEM (5 µM) for 48 h in the presence of CHX (35 µM) for the indicated times. (G, H) The effect of GEM on Tex10 degradation in HCT116 (G) or HT29 (H) cells as evaluated using lysosome (CQ, Baf A1) and proteasome (MG132) inhibitors. For A‐H, n = 3 biologically independent samples. E is presented as mean ± SD and analyzed by two‐sided Student's t test. Source data are provided as a Source data file.

### 3.7 Targeting of Tex10 Sensitizes CRC Cells to OXA In Vitro and In Vivo

To investigate the role of the Tex10 inhibitor GEM in modulating Tex10‐mediated OXA resistance, CRC cells were treated with GEM (500 nM) alone or in combination with OXA (30 µM) for 48 h. Apoptosis analysis by FACS showed that combined GEM and OXA treatment significantly increased cell sensitivity to OXA (Figure 8A, B). In HCT116 cells, co‐treatment with GEM (500 nM) and varying concentrations of OXA for 48 h was assessed using a CCK‐8 assay, which revealed that GEM reduced the OXA IC50 from 8.263 ± 0.554 µM to 3.389 ± 0.221 µM (Figure 8C). The synergistic effect of GEM on OXA sensitivity was further evaluated in vivo. Subcutaneous xenografts were established in Balb/c nude mice using HT29 cells. Once tumor volumes reached approximately 50 mm^3^, mice were randomly assigned to four groups receiving saline (control), OXA (2 mg/kg, every 3 days), GEM (30 mg/kg, every 3 days), or a combination of OXA + GEM for three weeks. Compared to OXA alone, GEM monotherapy significantly inhibited tumor growth, while the combination treatment further suppressed tumor progression, indicating a synergistic enhancement of OXA's antitumor efficacy (Figure 8D–F). IHC analysis demonstrated that, relative to single‐agent groups, the combination treatment resulted in lower expression of the proliferation marker Ki67 and higher expression of the apoptosis marker cleaved‐caspase 3 (Figure 8G,H). Importantly, no significant alterations in body weight, nor any overt toxicity or adverse effects, were observed in any treatment group (Figure S7A, B). To confirm whether GEM enhances OXA sensitivity via Tex10 targeting, patient‐derived CRC organoids were treated with GEM, OXA, or their combination for 4 days. Combination therapy markedly increased OXA sensitivity in these organoid models (Figure 8I–K). Collectively, these results demonstrate that GEM targets Tex10 to enhance OXA sensitivity both in vitro and in vivo.

**FIGURE 8:**
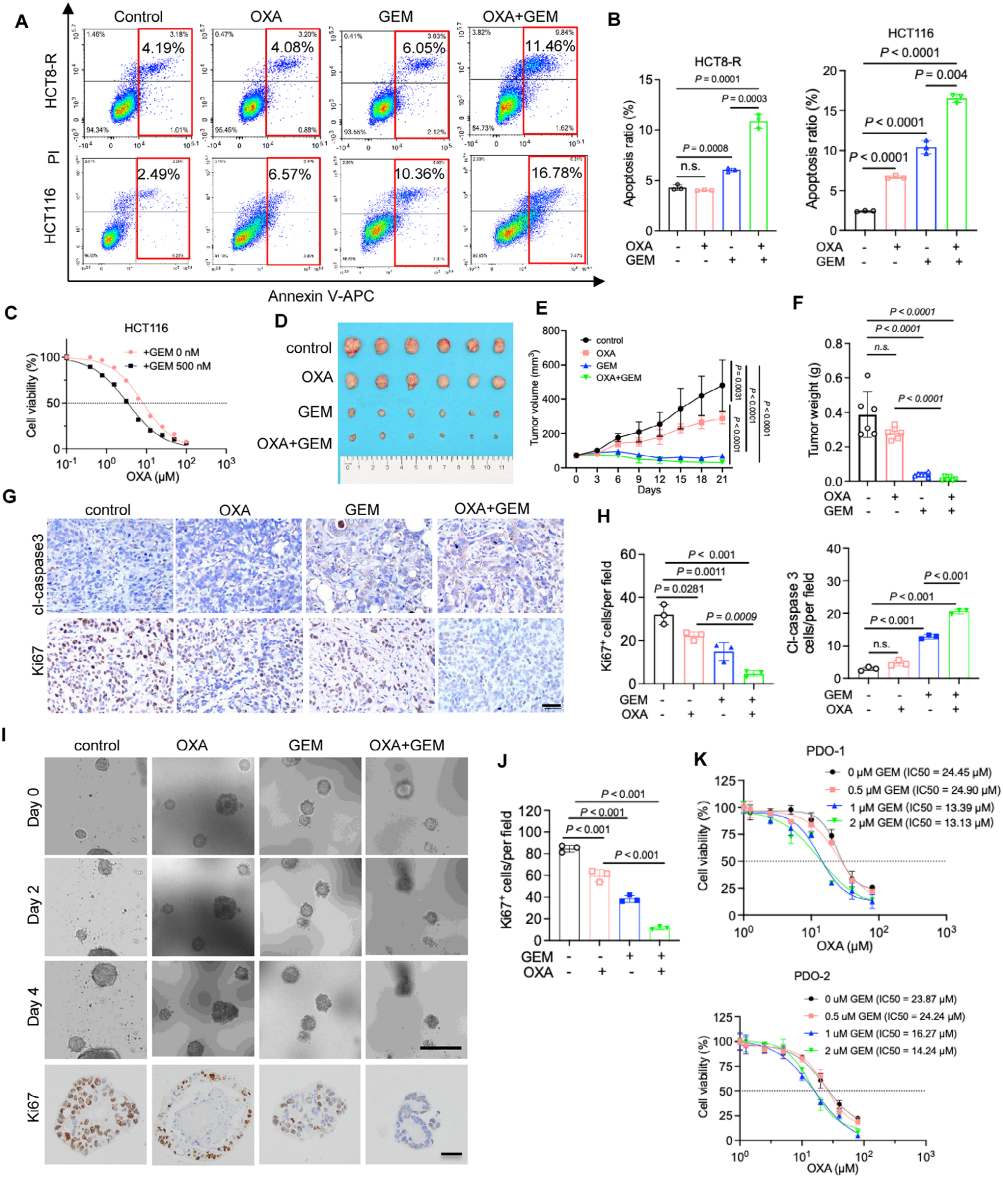
Targeting of Tex10 sensitizes CRC cells to OXA in vitro and in vivo. (A, B) The effect of GEM (1 µM) on apoptosis following OXA treatment (10 µM) for 24 h as detected by flow cytometry. (C)The IC50 of OXA in HCT116 cells subjected to GEM treatment. (D‐F) HT29 xenograft tumor model mice (n = 6 per group) were intraperitoneally administered vehicle, GEM (40 mg/kg), or OXA (3 mg/kg) every three days for three weeks. Tumor volumes (E) were measured every 3 days. After euthanizing the mice, tumors were excised, photographed (c), and weighed (F). (G, H) Immunohistochemical staining analysis of Ki67 levels in HT29 tumors. Scale bar:10 µm. (I, J) Representative bright‐field (BF) images showing the response of CRC organoids to OXA (30 µM) in the presence or absence of GEM (2 µM), together with IHC staining of Ki67 in organoids. Scale bar: 200 µm (upper) and 10 µm (bottom). (K) The IC50 of OXA in CRC organoids upon Tex10 depletion in the presence or absence of GEM. For A‐C, G, H, and I‐M, n = 3 biologically independent samples. For E and F, n = 6 biologically independent samples. Data are presented as mean ± SD. Data in C were analyzed using two‐sided Student's t tests. Data in B, E, F, H, and J were analyzed using one‐way ANOVAs. Source data are provided as a Source data file.

### 3.8 Targeting of Tex10 Inhibits PD‐L1 Degradation via a Lysosomal Pathway

Immunotherapy for CRC primarily targets PD‐1, and the PD‐1 inhibitor pembrolizumab has been approved as a first‐line treatment for CRC patients in the microsatellite instability (MSI) subgroup [35]. However, approximately 85% of CRC patients exhibit MSS and are thus unsuitable for current immunotherapy regimens [6, 36]. Given that autophagy plays a critical role in both chemotherapy resistance and immune escape and that targeting autophagy enhances immune checkpoint therapy, we investigated whether Tex10 influences PD‐L1 expression in CRC cells to explore its potential to improve immunotherapeutic efficacy [37, 38, 39]. Silencing Tex10 in both human CRC and mouse colon cancer cells upregulated total PD‐L1 expression (Figure 9A,C) and increased PD‐L1 surface expression (Figure 9B,D). Similarly, treatment of CRC cells with increasing concentrations of GEM upregulated PD‐L1 protein levels and elevated surface PD‐L1 expression in a dose‐dependent manner (Figure 9E,F). Interestingly, in HCT116 cells, both Tex10 silencing and GEM treatment reduced PD‐L1 mRNA levels (Figure 9G,H). Since Tex10 depletion significantly increased total and membrane‐bound PD‐L1 while decreasing PD‐L1 mRNA expression, we hypothesized that Tex10 regulates PD‐L1 protein stability post‐transcriptionally. As the proteasome and autophagy–lysosome systems are the two major pathways mediating protein degradation [40], we analyzed PD‐L1 protein half‐life using CHX chase assays. Compared to control cells, the half‐life of endogenous PD‐L1 protein was significantly prolonged in GEM‐treated cells, supporting the notion that Tex10 negatively regulates PD‐L1 protein stability (Figure 9I). To identify the degradation pathway responsible, RKO cells were treated with MG132 (proteasome inhibitor) or lysosomal inhibitors CQ and Baf A1. CQ and Baf A1, but not MG132, abolished the difference in PD‐L1 expression between GEM‐treated and untreated cells (Figure 9J). Notably, GEM increased PD‐L1 protein levels in control but not Tex10‐knockdown cells, indicating that GEM‐mediated PD‐L1 upregulation is largely Tex10‐dependent (Figure 9K). Together, these findings demonstrate that Tex10 primarily regulates PD‐L1 protein stability through a lysosome‐dependent degradation pathway.

**FIGURE 9:**
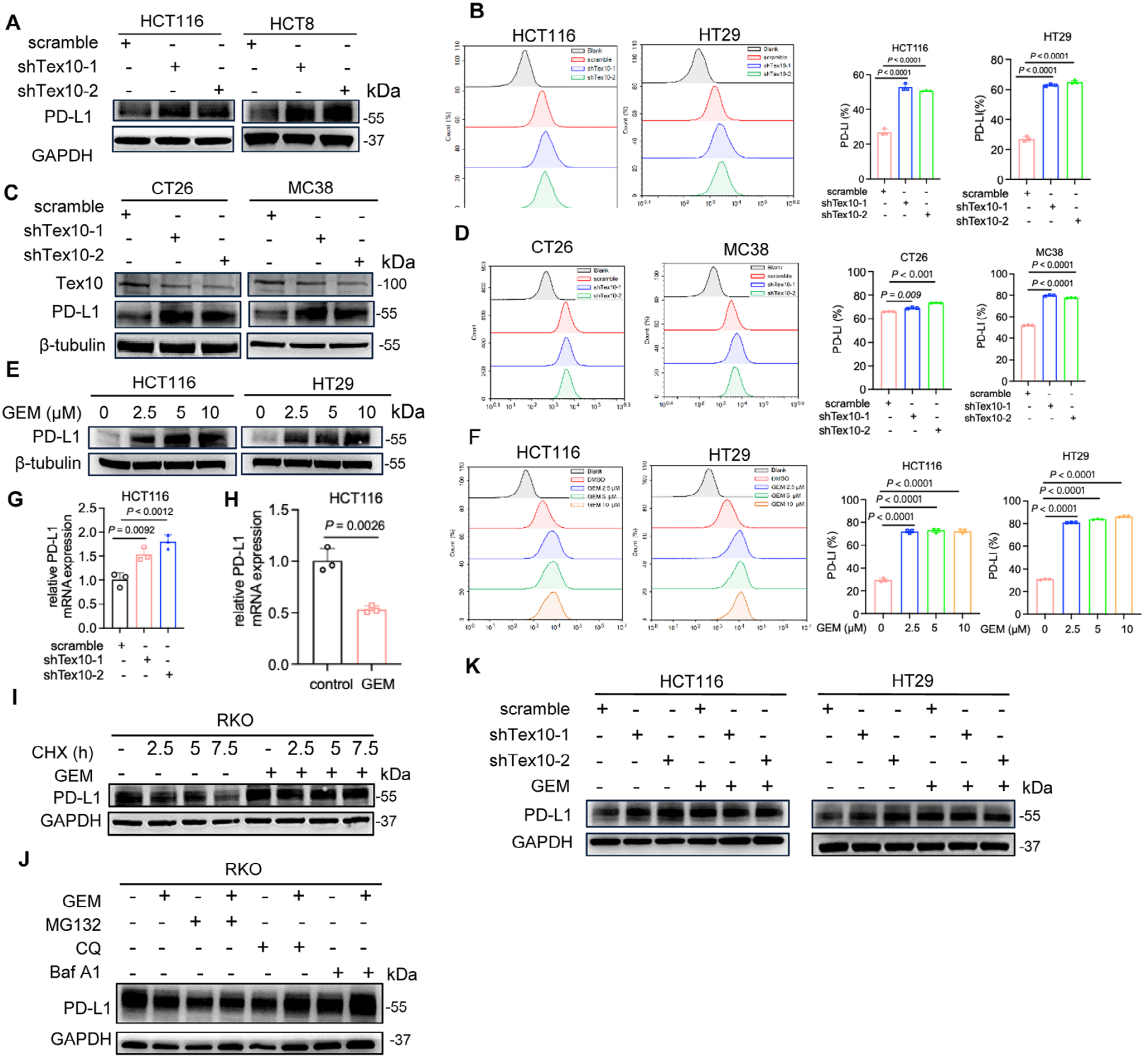
Deletion of Tex10 inhibits the lysosome‐dependent degradation of PD‐L1. (A) Immunoblotting analysis of PD‐L1 expression in the indicated CRC cells stably expressing scramble or shTex10 constructs. (B) Flow cytometry analysis of PD‐L1 expression in Tex10‐silenced HCT116 and HT29 cells. (C) Immunoblotting analysis of PD‐L1 expression in the indicated mouse colon cancer cells transfected with shTex10 plasmids. (D) Flow cytometry analysis of PD‐L1 expression in Tex10‐silenced CT26 and MC38 cells. (E) Immunoblotting analysis of PD‐L1 expression in HCT116 and HT29 cells treated with the indicated concentrations of GEM for 48 h. (F) HCT116 and HT29 cells were treated with GEM (0, 2.5, 5, and 10 µM) for 48 h, and plasma membrane PD‐L1 was detected by flow cytometry. (G) qRT‐PCR analysis of the change of PD‐L1 caused by targeting Tex10 with shRNA. (H) qRT‐PCR analysis of the change in PD‐L1 expression caused by Tex10 overexpression. (I) Immunoblotting analysis of PD‐L1 abundance in RKO cells treated with DMSO or GEM (5 µM) for 48 h in the presence of CHX (35 µM) for the indicated times. (J) Immunoblotting analysis of PD‐L1 abundance in RKO cells treated with GEM (5 µM) for 48 h in the presence of MG132, CQ, or Baf A1. (K) Immunoblotting analysis of PD‐L1 in HCT116 and HT29 cells stably expressing scramble or shTex10 constructs and treated with or without GEM (5 µM) for 48 h. For A‐I, n = 3 biologically independent samples. For A‐J, n = 3 biologically independent samples. Data are presented as mean ± SD. Data in B, D, F, and G were analyzed using one‐way ANOVAs. Data in H were analyzed by two‐sided Student's t test. Source data are provided as a Source data file.

### 3.9 Targeting of Tex10 Enhances the Antitumor Effect of Immunotherapy

To evaluate the impact of Tex10 inhibition on anti‐PD‐1/PD‐L1 immunotherapy in vivo, we established a CT26 tumor model in immunocompetent BALB/c mice. Our results demonstrated that depletion of Tex10 sensitized tumors to anti‐PD‐1 and anti‐PD‐L1 therapies (Figure 10A–C). Analysis of infiltrating immune cells revealed that Tex10 silencing significantly increased the proportion of CD8^+^ T cells, while combination therapy with either anti‐PD‐1 or anti‐PD‐L1 antibodies further enhanced CD8^+^ T‐cell infiltration (Figure 10D). To assess the activation status of tumor‐infiltrating CD8^+^ T cells, we examined the expression of Granzyme B (GzmB), a cytotoxic T‐cell activation marker. GzmB expression was significantly upregulated following Tex10 silencing or treatment with anti‐PD‐1/anti‐PD‐L1 (Figure 10E). Furthermore, the proportion of regulatory T cells (CD4^+^CD25^+^Foxp3^+^) was decreased in Tex10‐deficient tumors treated with control IgG, anti‐PD‐1, or anti‐PD‐L1, suggesting that Tex10 depletion reprograms the tumor immune microenvironment from an immunosuppressive to an immune‐activated state (Figure 10F). Consistent with the phenotype observed following Tex10 depletion, pharmacological inhibition of Tex10 with GEM significantly suppressed tumor growth in vivo. Although monotherapy with GEM, anti‐PD‐1, or anti‐PD‐L1 each suppressed tumor growth, combination regimens comprising GEM plus either anti‐PD‐1 or anti‐PD‐L1 yielded superior reductions in tumor volume, weight, and growth rate compared with controls (Figure 10G–I). Furthermore, no significant alterations in body weight, nor any overt toxicity or adverse effects, were observed in any treatment group (Figure S8A, B). Collectively, these findings support the conclusion that Tex10 plays a critical role in shaping the tumor immune microenvironment and that disrupting Tex10‐mediated PD‐L1 regulation is key to potentiating the efficacy of anti‐PD‐1/PD‐L1 immunotherapies.

**FIGURE 10:**
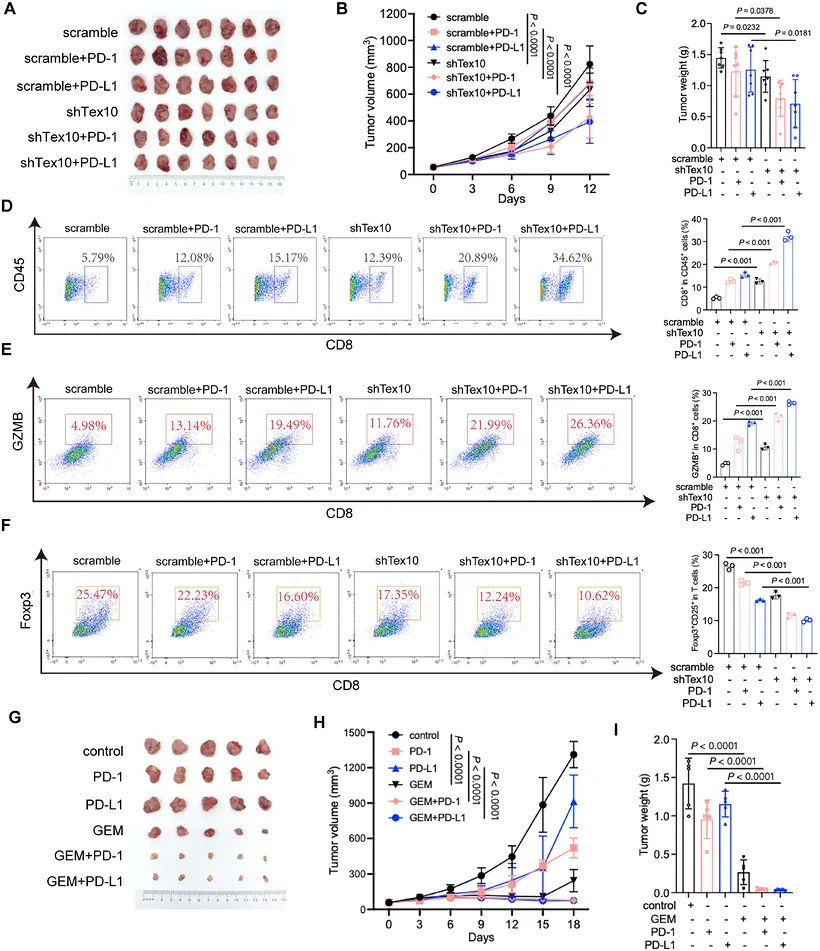
Depletion of Tex10 improves the efficacy of immune checkpoint inhibition. CT26 tumors formed by control and Tex10 knockdown cells were established orthotopically in BALB/c mice that received control IgG, anti‐PD‐L1, or anti‐PD‐1 treatment. (A‐C) CT26 tumor images (A), tumor volume (B), and tumor weight (C) were measured for 12 days. (D) Flow cytometry analysis of CD8^+^T cells among tumor‐infiltrating lymphocytes in CT26 tumors in the indicated treatment groups. (E, F) Flow cytometry analysis of GzmB^+^ CD8^+^ (E) and Foxp3^+^CD25^+^ CD4^+^ (F) cells in the CT26 tumors from the indicated treatment groups. (G‐I) BALB/c mice bearing CT26 cells were treated with control IgG, anti‐PD‐L1, anti‐PD‐1, GEM, GEM + anti‐PD‐L1, and GEM + anti‐PD‐1. CT26 tumor images (G), tumor volume (H), and tumor weight (I) were measured for 18 days. For B and C, n = 7 biologically independent samples. B‐F are presented as mean ± SD and analyzed using one‐way ANOVAs. For H and I, n = 5 biologically independent samples. H and I are presented as mean ± SD and analyzed using one‐way ANOVAs. Source data are provided as a Source Data File.

## 4 Discussion

OXA‐based regimens, including FOLFOX and CAPOX, represent the standard adjuvant chemotherapy for postoperative CRC. The OXA‐based FOLFOX regimen, often combined with targeted agents such as bevacizumab or cetuximab, is the first‐line treatment for advanced or metastatic CRC and also serves as a principal neoadjuvant strategy for locally advanced disease. However, acquired chemoresistance following prolonged OXA exposure remains virtually inevitable, presenting a major therapeutic challenge. Despite significant clinical relevance, the molecular mechanisms underlying OXA resistance in CRC remain incompletely defined. This study addresses this gap by identifying Tex10 as a key regulator of OXA resistance in CRC and demonstrating the therapeutic potential of targeting Tex10 to overcome this resistance. We found that Tex10 competitively binds BRD9, thereby disrupting BRD9‐BRG1 interactions within the ncBAF complex. This disruption suppresses AMBRA1‐mediated ULK1 ubiquitination and autophagy in CRC cells. Moreover, pharmacological inhibition of Tex10 with the FDA‐approved agent GEM restores OXA sensitivity in vitro and in vivo, while Tex10 deletion enhances antitumor immunity and synergizes with immune checkpoint blockade. Notably, elevated Tex10 expression in biopsy specimens correlates with poor prognosis and may serve as a predictive biomarker for patient response to OXA‐based chemotherapy.

Previous studies have established Tex10 as a key regulator of pluripotency and self‐renewal in mouse embryonic stem cells [10] and as a factor involved in spermatogenesis [12]. Tex10 is also a component of the ribosomal core scaffold (PELP1‐WDR18‐TEX10‐LAS1L) that regulates ribosome biogenesis [13]. Mounting evidence implicates Tex10 in promoting tumor progression, chemoresistance, and metastasis across multiple cancer types [14, 15, 41]. Our prior research demonstrated that Tex10 facilitates metastasis in hepatocellular carcinoma and promotes EMT in esophageal squamous cell carcinoma [16, 42]. However, its role in OXA resistance has remained unexplored. In this study, we found that high Tex10 expression is associated with poor prognosis in CRC and is elevated in patients who have developed OXA resistance following OXA‐based chemotherapy. OXA exposure dose‐dependently increased Tex10 expression in CRC cells, and OXA‐resistant cell lines display constitutively high Tex10 levels. Functional validation experiments confirmed that Tex10 enhances OXA resistance both in vitro and in vivo, establishing Tex10 as a critical driver of acquired chemoresistance in CRC.

Autophagy is a conserved self‐degradative process essential for maintaining cellular homeostasis and can exert either tumor‐suppressive or tumor‐promoting effects depending on context [43, 44]. Clinical trials have validated the safety of autophagy inhibition using hydroxychloroquine, though efficacy remains suboptimal [22, 45, 46, 47]. As new autophagy regulators enter clinical evaluation, understanding their mechanistic contributions to therapy resistance becomes increasingly important. Given the complex interplay between autophagy and tumor progression, we investigated the involvement of autophagy in Tex10‐mediated OXA resistance. Our data demonstrate that Tex10 suppresses autophagic flux and autophagosome formation in CRC cells. Among the four major autophagy modalities, including cytoprotective, cytotoxic, cytostatic (growth‐inhibitory), and nonprotective autophagy [48], Tex10 silencing induces cytostatic autophagy, which in turn critically primes the cells for enhanced OXA‐induced cytotoxic cell death.

To elucidate the underlying mechanism in greater detail, we examined key upstream regulators of autophagy initiation, including the energy sensor AMPK and the nutrient sensor mTORC1, both of which control ULK1 complex activity [21]. We found that Tex10 regulates autophagy independently of the canonical AMPK‐mTOR axis, instead suppressing ULK1 protein stability by reducing its K63‐linked ubiquitination. Consistent with previous findings that AMBRA1 promotes autophagy via ULK1 ubiquitination and activation [31], we showed that Tex10 modulates ULK1 and p62 expression in an AMBRA1‐dependent manner, thereby controlling CRC autophagy through AMBRA1 regulation. Furthermore, BRG1, which is a key subunit of the SWI/SNF chromatin remodeling complex, has been shown to transcriptionally regulate Ambra1 expression, maintaining intestinal epithelial homeostasis and preventing inflammation and tumorigenesis [32]. BRG1 is also essential for CRC stem cell maintenance [49]. Our study reveals that Tex10 inhibits BRG1‐mediated transcription of AMBRA1, and that BRG1 silencing in Tex10‐depleted CRC cells reverses AMBRA1 upregulation and restores p62 expression. Collectively, these findings establish that Tex10 orchestrates autophagy in CRC cells via the BRG1/AMBRA1 axis.

To further delineate the molecular mechanism underlying Tex10‐mediated regulation of BRG1/AMBRA1‐dependent autophagy in CRC cells, we performed co‐immunoprecipitation coupled with mass spectrometry (IP‐MS), which identified BRD9 as a novel Tex10‐interacting partner. Because BRD9 assembles with the ATPase BRG1 within the ncBAF complex, we hypothesized that Tex10 binding to BRD9 might disrupt the complex's role in autophagy regulation. Specifically, we examined whether Tex10 competitively binds BRD9 to interfere with BRD9‐BRG1 subunit interactions, thereby suppressing autophagy. Our findings revealed that BRD9‐BRG1 binding was attenuated in the presence of Tex10. Tex10 interacts with the DUF domain of BRD9, whereas BRG1 engages both the N‐terminal and DUF domains of BRD9. Thus, Tex10 competitively binds to the BRD9 DUF domain, directly competing with BRG1. Notably, Tex10‐BRD9 binding was disrupted under autophagy‐inducing conditions, restoring ncBAF‐mediated positive regulation of autophagy. These results demonstrate that Tex10 suppresses AMBRA1 transcription‐dependent ULK1 ubiquitination and autophagy by competitively binding BRD9 and destabilizing BRD9‐BRG1 interactions within the ncBAF complex. Furthermore, we established that BRG1‐dependent transcriptional regulation of AMBRA1‐mediated autophagy in CRC cells requires BRD9 participation.

Here, we identified GEM as a small‐molecule inhibitor of the Tex10 protein. Binding affinity and specific interaction sites, particularly at residue N268, were validated using SPR, CETSA, and Lip/MS. As a deoxynucleoside analog, GEM conventionally inhibits DNA synthesis to induce apoptosis and is widely used either as monotherapy or in combination regimens for first‐line treatment of various solid tumors, including advanced pancreatic cancer and intrahepatic cholangiocarcinoma [50, 51]. However, GEM has not been incorporated into systemic therapy for CRC due to intrinsic chemoresistance, rapid metabolic inactivation (e.g., by cytidine deaminase), and lack of patient stratification [52]. Critically, we discovered that GEM exerts autophagy‐regulatory effects in a Tex10‐dependent manner and promotes lysosome‐dependent autophagic degradation of Tex10. Using the Tex10‐N268A mutant, we confirmed that this autophagic effect is driven by specific on‐target engagement rather than nonspecific DNA synthesis inhibition or general cytotoxicity. Because autophagy plays an essential role in modulating tumor immunity, we further explored GEM's immunoregulatory potential. Combination regimens of GEM with immune checkpoint inhibitors (e.g., nivolumab or atezolizumab) are currently being evaluated in clinical trials for pancreatic cancer, urothelial carcinoma, and biliary tract cancers [53, 54, 55]. In contrast, for CRC, pembrolizumab (anti‐PD‐1) is approved only for the approximately 15% of patients exhibiting MSI, whereas the remaining 85%, classified as MSS, are generally resistant to ICB therapy [5]. Thus, novel strategies to enhance immunotherapy efficacy in MSS CRC are urgently needed. We found that GEM upregulates PD‐L1 surface expression in CRC cells and enhances PD‐L1 stability via lysosome‐dependent mechanisms. This raises a key question: is the stabilized PD‐L1 functional? Our in vivo experiments demonstrated that GEM combined with PD‐1 or PD‐L1 antibodies markedly enhances ICB efficacy. Critically, this immune activation depends on blocking antibodies, demonstrating that the stabilized PD‐L1 can engage PD‐1 in vivo. Thus, Tex10 inhibition preserves PD‐L1 function while sensitizing tumors to ICB. Mechanistically, Tex10 inhibition primes tumors for ICB by stabilizing PD‐L1, suggesting that pharmacological PD‐L1 upregulation via Tex10 targeting can convert ICB‐resistant MSS CRC into a vulnerable state.

We observed a strong correlation between elevated Tex10 expression and poor response to OXA‐based therapy in CRC patients, suggesting that pre‐treatment. assessment of Tex10 in biopsy specimens may facilitate personalized therapeutic strategies for patients with advanced CRC. Patients with high Tex10 expression may benefit from GEM combined with OXA‐based chemotherapy or immunotherapy, as our findings establish that GEM enhances OXA sensitivity and improves response to immune checkpoint blockade by specifically targeting Tex10. Nevertheless, several challenges must be acknowledged. GEM is not standard for CRC, and previous clinical studies in unselected populations showed limited efficacy due to intrinsic resistance, metabolic inactivation, and lack of biomarker‐guided selection. Additionally, GEM may exert Tex10‐independent effects via DNA synthesis inhibition, though our CETSA, SPR, LiP‐MS, and N268‐mutagenesis data support a specific Tex10‐dependent mechanism without excluding parallel canonical GEM activities. While low‐dose GEM was well tolerated in our mouse models, comprehensive pharmacokinetic and toxicity evaluations remain necessary. Beyond these drug‑related considerations, additional experimental limitations warrant mention: the in vitro studies on GEM‐mediated OXA sensitization require validation in additional patient‐derived organoid models; the docking model is based on an AlphaFold‐predicted structure rather than an experimentally resolved one. While our orthogonal biochemical data (CETSA, SPR, and LiP‐MS) collectively support direct binding of GEM to Tex10 at N268, definitive validation of the binding interface will ultimately require a co‐crystal structure or NMR analysis. Given the novelty of this binding pocket, we are actively pursuing crystallization of the Tex10–GEM complex to address this limitation in future work; the in vivo experiments should be expanded to include patient‐derived xenograft models to strengthen translational relevance; studies examining GEM‐enhanced immunotherapy sensitivity will require larger animal cohorts, additional preclinical validation, and eventual clinical evaluation; and finally, the relatively small clinical cohort limits definitive conclusions regarding Tex10's predictive value for OXA response, which should be prospectively assessed in larger, independent CRC populations.

## 5 Conclusions

Our findings demonstrate that elevated Tex10 expression is correlated with OXA resistance in CRC. Tex10 promotes OXA resistance primarily by suppressing cytostatic autophagy. Mechanistically, Tex10 competitively binds to BRD9, disrupting BRG1‐BRD9 interactions within the ncBAF complex, thereby inhibiting AMBRA1 transcription‐dependent ULK1 ubiquitination and autophagic initiation, ultimately driving OXA chemoresistance in CRC. Leveraging the identification of GEM as a Tex10 inhibitor, the GEM/OXA combination regimen represents a promising therapeutic strategy to overcome OXA resistance. Moreover, GEM plays a vital role in enhancing anti‐PD‐1 efficacy in CRC by stabilizing PD‐L1 and promoting antitumor immune activation.

## Author Contributions

**Ping Xu**: Writing – original draft, Data curation. **Jie Huang**: Data curation, Investigation. **Xiaojin Gao**: Validation, Methodology, Formal analysis. **Lin Deng**: Validation, Methodology. **Yanheng Deng**: Investigation, Methodology. **Denglu Wang**: Validation, Methodology. **Chunlei Yu**: Software, Methodology. **Yunxiang Zhang**: Data curation, Supervision, Project administration, Methodology. **Jingdong Li**: Supervision, Project administration, Funding acquisition, Conceptualization. **Xiaocong Xiang**: Writing – review & editing, Supervision, Project administration, Funding acquisition, Data curation, Conceptualization.

## Conflicts of Interest

The authors declare no conflict of interest.

## Supporting information




**Supporting File 1**: advs76895‐sup‐0001‐SuppMat1.docx.

**Supporting File 2**: advs76895‐sup‐0002‐DataSet.zip.

## Data Availability

The TCGA dataset used in this study is publicly available from TCGA. The remaining data are available within the Article, Supplementary information, or Source Data file. Source data are provided with this paper.
