# Supplementary material for: Targeting Tex10 Overcomes Oxaliplatin Resistance by Competitively Disrupting the Non‐Canonical BAF Complex in Colorectal Cancer
Source: Adv Sci (Weinh). 2026 Jul 29:e76895. Online ahead of print. doi: 10.1002/advs.76895 (PMC13418743; doi:10.1002/advs.76895)
Supplement: Supplementary file 1 — Supporting File 1: advs76895‐sup‐0001‐SuppMat1.docx. [file ADVS-9999-e76895-s001.docx]

**Supplementary information**

**Targeting Tex10 overcomes oxaliplatin resistance by competitively disrupting the non-canonical BAF complex in colorectal cancer**

**Supplementary figures**


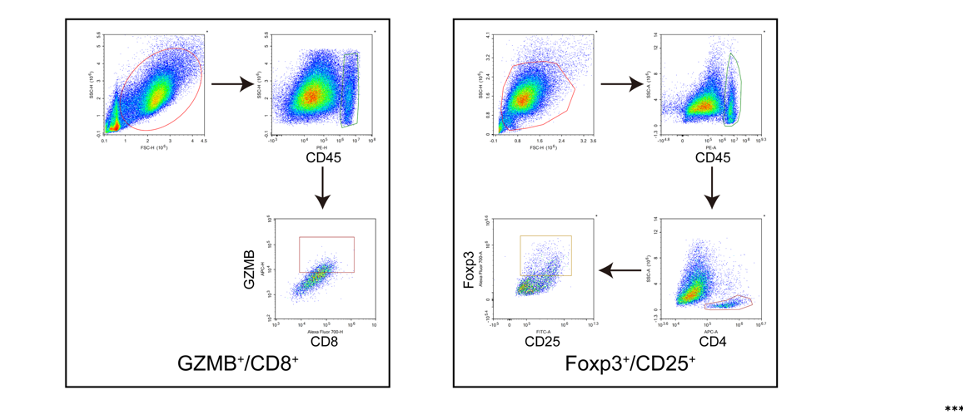


**Supplementary Figure 1.** Gating strategy for Granzyme B and Foxp3 expression measured by flow cytometry.

**
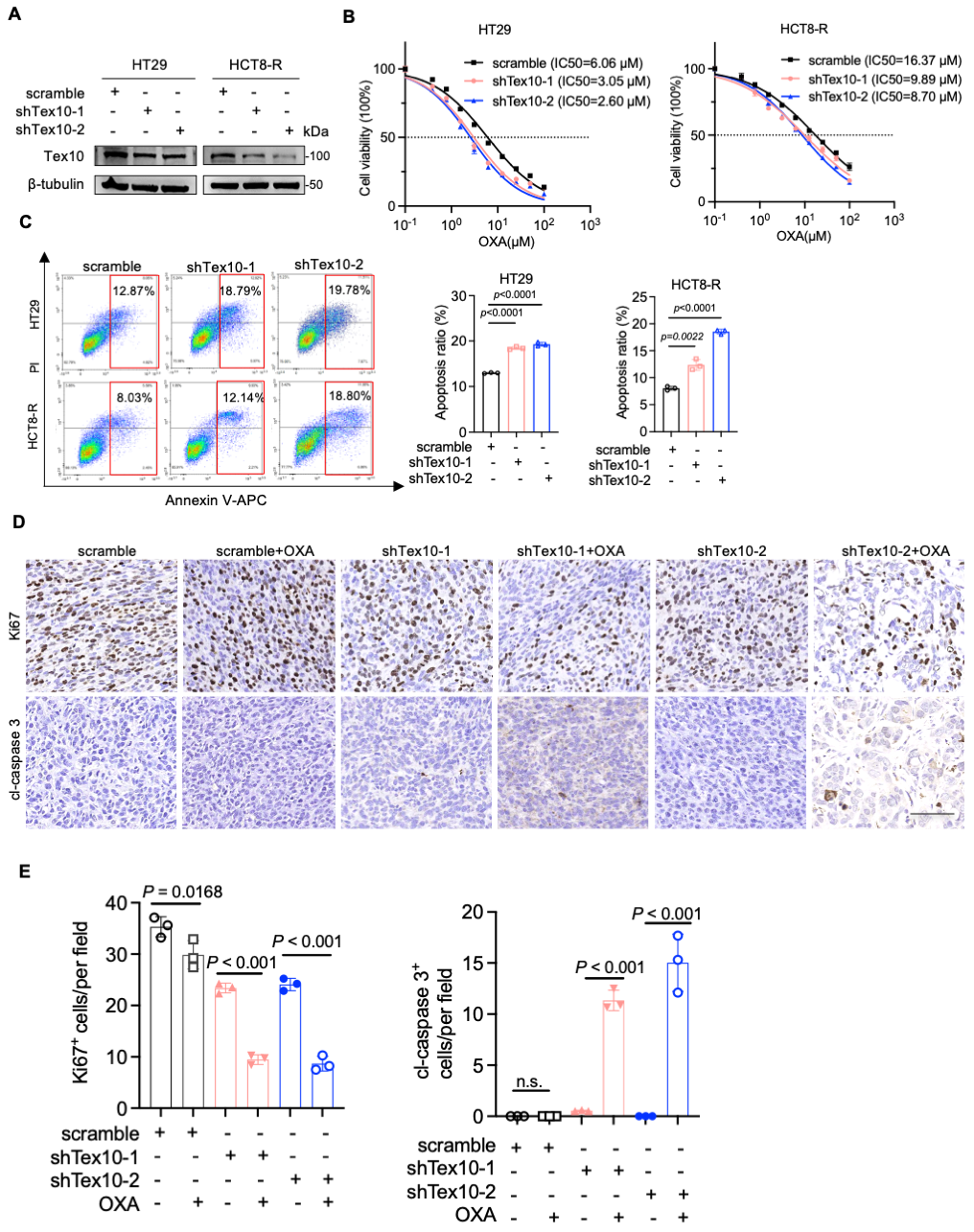
**

**Supplementary Figure 2.** Tex10 promotes OXA resistance in vitro and in vivo. (A) Immunoblotting analysis of the knockdown efficiency of Tex10 in CRC cells. (B) Sensitivity to OXA in scramble (negative control shRNA) and shTex10 (Tex10-targeted shRNA) cells. (C) The effect of Tex10 silencing on apoptosis following OXA treatment (30 μM, 48 h) as detected by flow cytometry. (D, E) IHC staining for Ki67 and cleaved Caspase 3 in xenograft tumor from subcutaneous implanted shTex10 HT29 cells and control cells treated with OXA or PBS. Scale bars, 50 μm. Quantification of IHC staining is shown below (E). For A and D, n = 3 biologically independent samples. B，C and E are presented as mean ± SD and analyzed using one-way ANOVAs. Source data are provided as a Source Data file.


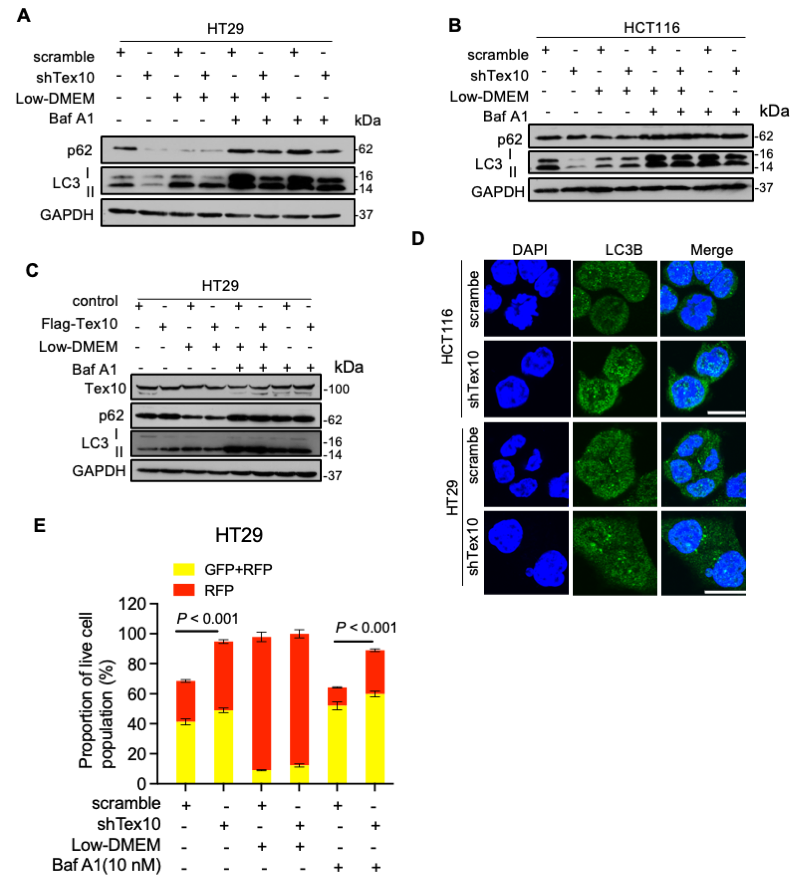


**Supplementary Figure 3.** Tex10 inhibits autophagic vesicle formation and autophagic flux. (A-C) Western blotting analysis of LC3 and p62 in Tex10 WT, shTex10 or Tex10-overexpressing cells after Low-DMEM (12 h) or Baf A1 (10 nM, 12 h) treatment. (D) Representative confocal images of LC3B in HCT116 and HT29 cells; the nucleus was stained with DAPI. Scale bar: 10 μm. (E) HT29 cells stably expressing shTex10 or control vector were transfected with GFP-mRFP-LC3 cultured in complete or starvation medium treated with or without Baf A1 (10 nM) for 12 h, and the expression of GFP and RFP was detected by flow cytometry. For A-D, n = 3 biologically independent samples. Data in E were analyzed using one-way ANOVAs. Source data are provided as a Source data file.

**
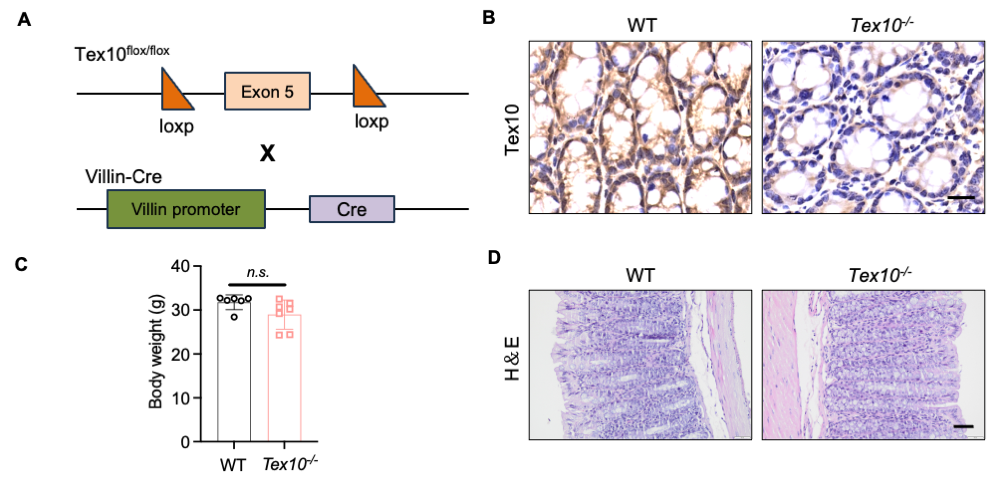
**

**Supplementary Figure 4.** Construction of Tex10 conditional knockout mice. (A) Schematic illustration of the knock-out region of Tex10^-/-^ mice. (B) Immunohistochemical of Tex10 expression are shown. Scar bar: 20 μm. (C) Body weight of 8-week-old Tex10^-/-^ mice (n =6) compared with WT littermates (n = 7). (D) Representative images of H&E-stained colonic sections from WT and Tex10^-/-^ mice at 2 months of age (n = 6 per genotype). Scale Bars: 500 μm. For C is presented as mean ± SD and analyzed using two-sided Student’s t tests. Source data are provided as a Source Data file.

**
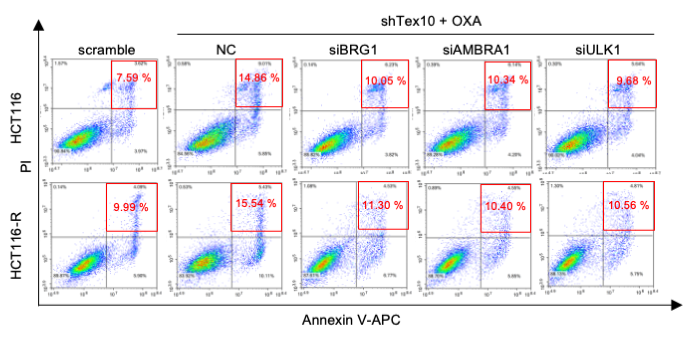
**

**Supplementary Figure 5.** BRG1/AMBRA/ULK1 is required for OXA resistance. The effect of AMBRA1, BRG1 or ULK1 silence on apoptosis as shown by flow cytometry analysis of Annexin V staining in Tex10-knockdown HCT116 and HCT116-R cells treated with OXA (40 μM).

a


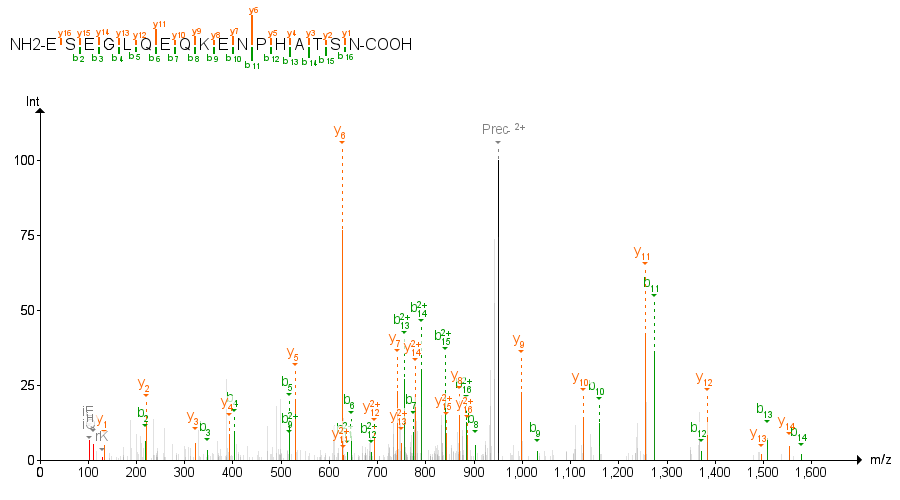


247-ESEGLQEQKENPHATSN-263

b


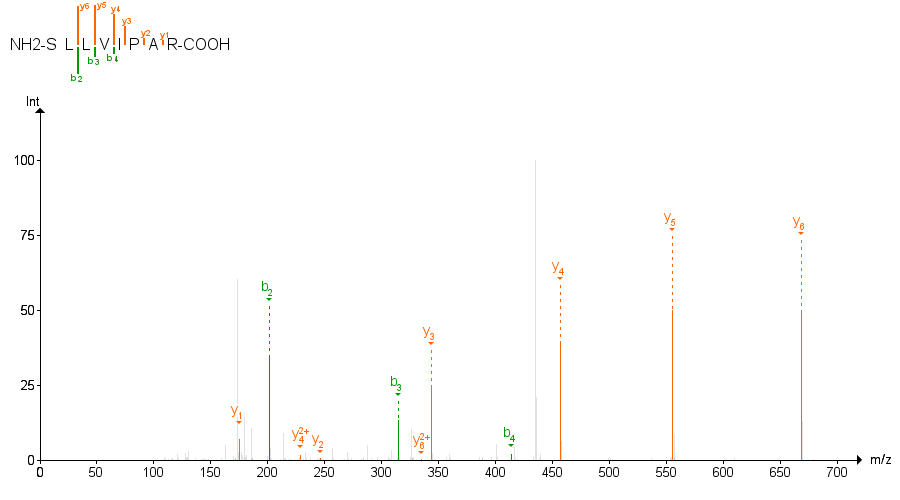


739-SLLVIPAR-746

c


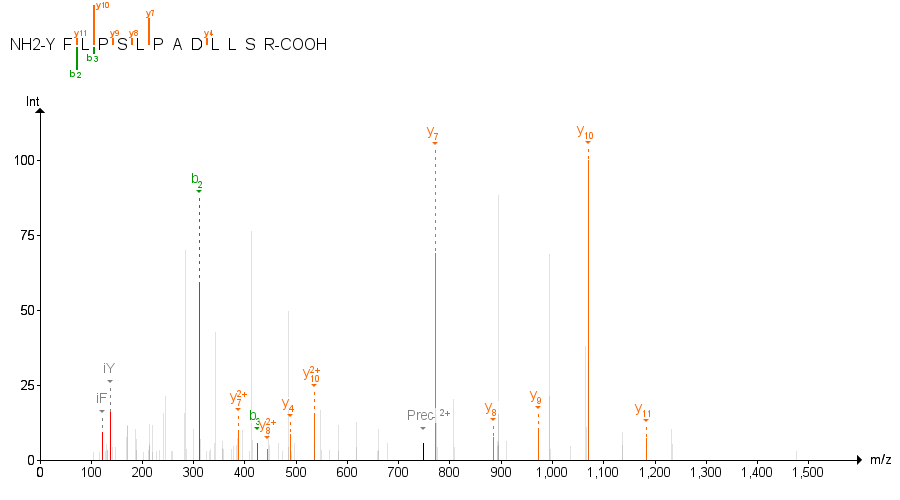


623-YFLPSLPADLLSR-635

**Supplementary Figure 6.** Identification of modification sites by LC-MS/MS. Recombinant Tex10 was incubated with GEM or DMSO and was then digested with Proteinase K, then the protein was digested with trypsin and analyzed by LC-MS/MS. The data was processed in FragPipe v 22.0.

**
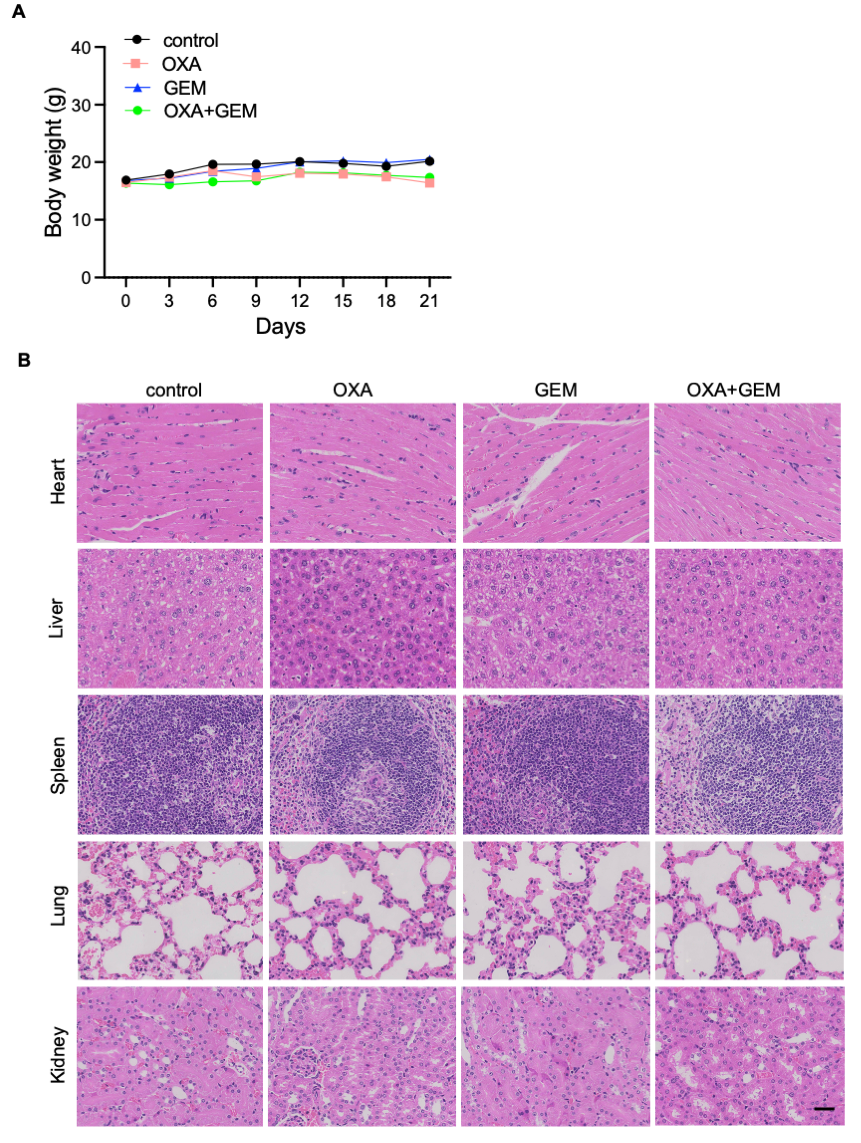
**

**Supplementary Figure 7.** No obvious toxic side effects of OXA and Tex10 inhibitor (GEM) combination therapy in mice. (A) Body weight of mice in the indicated groups was monitored. (B) Representative hematoxylin and eosin (H&E) staining of major organs from C57BL/6 mice in the indicated groups (n = 3). Scale bar, 25 μm.

**
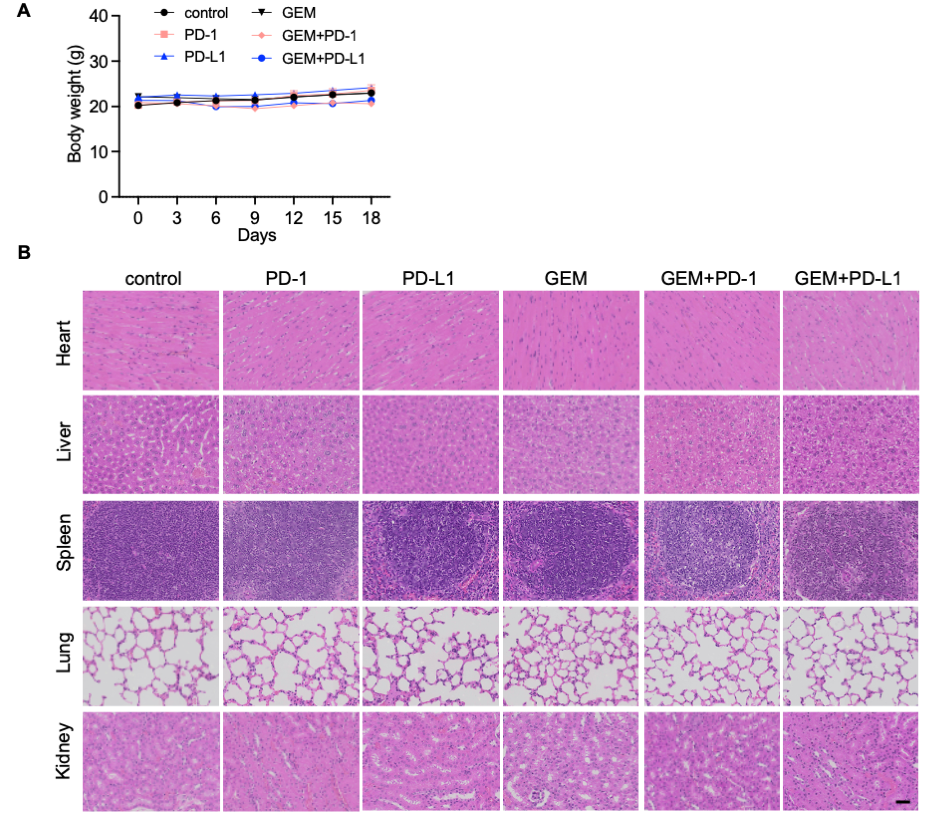
**

**Supplementary Figure 8.** No obvious toxic side effects of GEM combined with anti-PD-1/PD-L1 antibody in mice. (A) Body weight of mice in the indicated groups. (B) Representative H&E staining of major organs from C57BL/6 mice in the indicated groups (n = 3). Scale bar, 25 μm.

**Supplementary Table 1. Sequences of RNA Oligonucleotides**

| **Names** | **siRNAs (5’-3’)** |
| --- | --- |
| **siRNA** |  |
| siAMBRA1-1 | CAGTGAGAACAACTCCAAC |
| siAMBRA1-2 | TGGTGAAGACAGCTAGTGA |
| siBRG1-1 | UCUCCGUCAGUGAGUCGCU |
| siBRG1-2 | UCUCUAGGUCGUUGAGGCU |
| siULK1 | CGGAGAGCCUGCAGGAGAA |
| **shRNA** |  |
| shTex10-1 (human) | AGCTACTGCCCTCCGAATTTA |
| shTex10-2 (human) | ATGAGTGATGTAGACTATTTC |
| shTex10-1（mouse） | GGATGGAGTCACTCGGTTAAT |
| shTex10-2（mouse） | GATAGAACACTTCCGACAAAC |

**Supplementary Table 2. The primers used in present study**

| **Primer names** | **Forward** **(5’-3’)** | **Reverse (5’-3’)** |
| --- | --- | --- |
| Tex10 | TTGCAACTTGCTCATCTTGG | AGAGTCTGCAGGGAGAACCA |
| BECN1 | AGACCCAGGAGGAAGAGACT | AGCTGTTGGCACTTTCTGTG |
| ULK1 | GGCAAGTTCGAGTTCTCCCG | CGACCTCCAAATCGTGCTTCT |
| AMBRA1 | AACCCTCCACTGCGAGTTGA | TCTACCTGTTCCGTGGTTCTCC |
| PD-L1 | TGGCATTTGCTGAACGCATTT | TGCAGCCAGGTCTAATTGTTTT |

**Supplementary Table 3. The chip-qPCR primers used in present study**

| **Primer names** | | **Forward (5’-3’)** | **Reverse (5’-3’)** |
| --- | --- | --- | --- |
| AMBRA1-M1 | 5’-GGTGTGATCATGGCTCATCG-3’ | | 5’-TGAGCCCTGGAGTTCAAGAC-3’ |
| AMBRA1-M2 | 5’-ACCCGTGGCTTATGATCTGG-3’ | | 5’-ATACAAGAACGCCCCAGACA-3’ |
| AMBRA1-M3 | 5’-ATTCCCCTCACTGCTCTGTT-3’ | | 5’-CGCCGGCTGTTAAAGTTCTT-3’ |
