# Supplementary material for: Targeting Tex10 Overcomes Oxaliplatin Resistance by Competitively Disrupting the Non‐Canonical BAF Complex in Colorectal Cancer
Source: Adv Sci (Weinh). 2026 Jul 29:e76895. Online ahead of print. doi: 10.1002/advs.76895 (PMC13418743; doi:10.1002/advs.76895)

Unprocessed western blots

Figure 1H

=Used

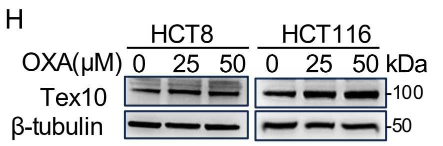

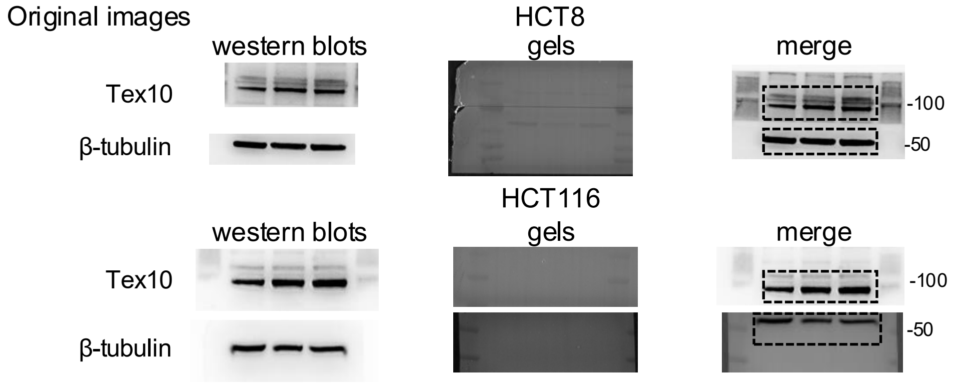

Figure 1I

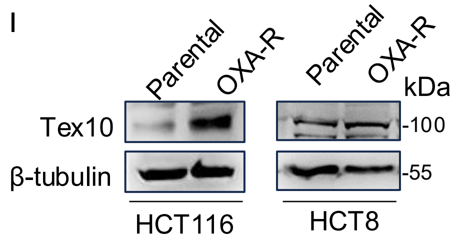

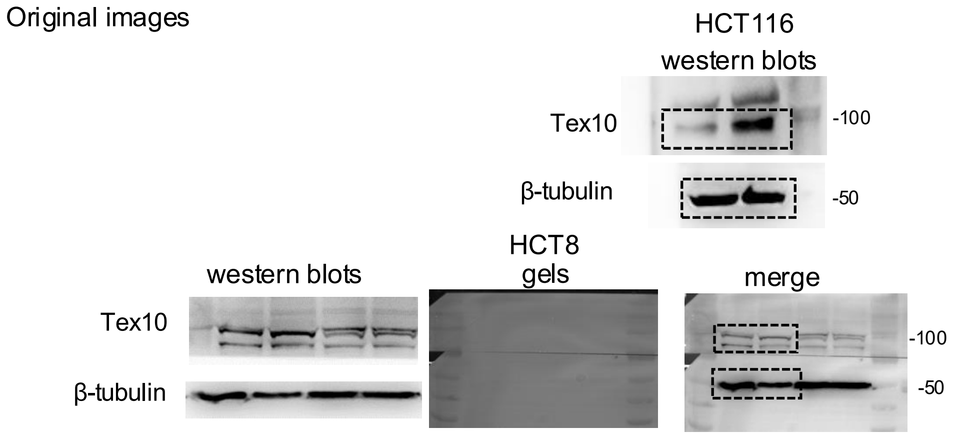

Figure 2A

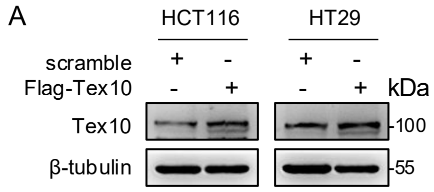

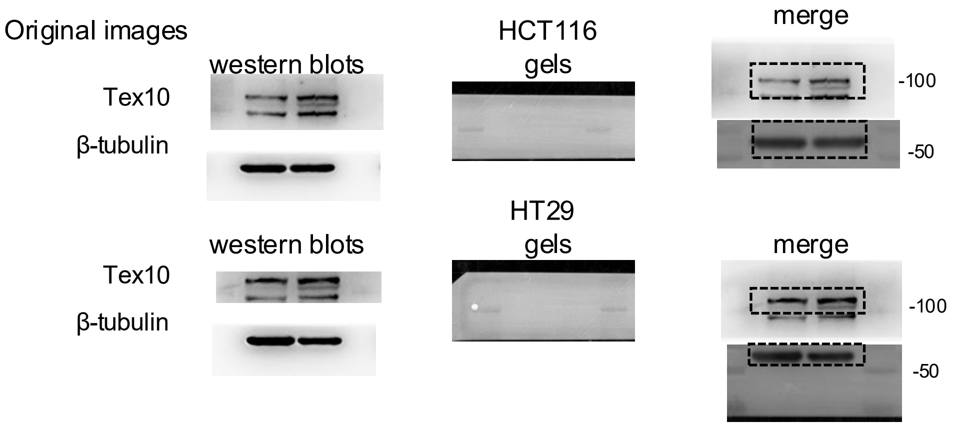

Figure 2D

=Used

Original images

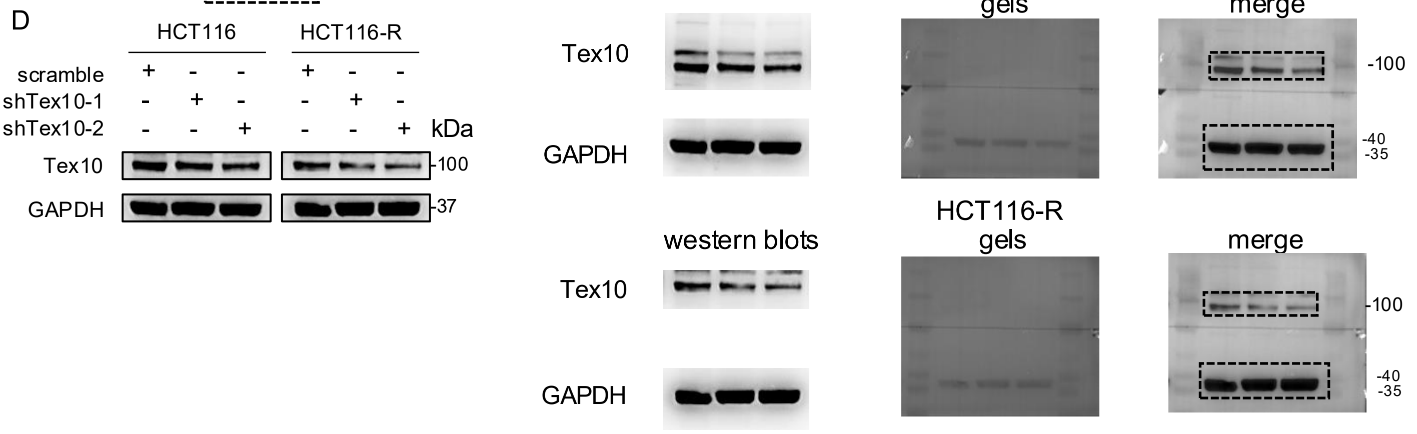

Figure 3A

Original images

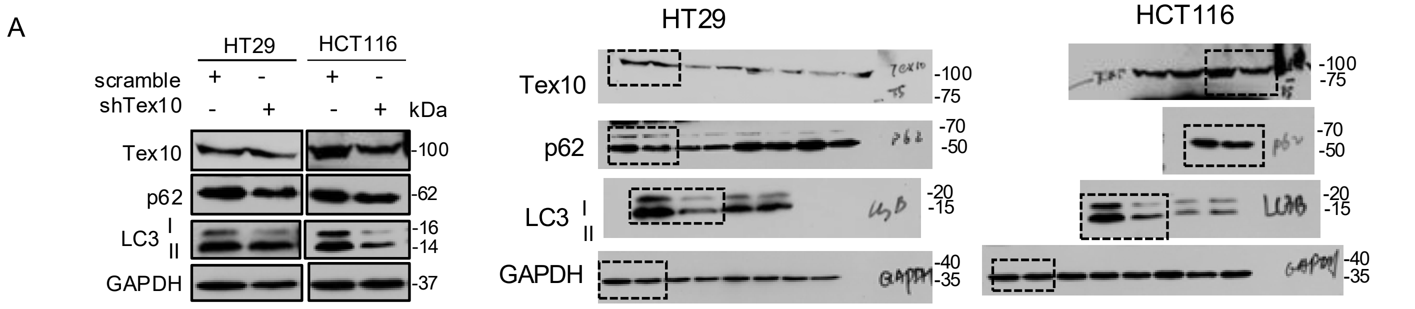

Figure 3B

Original images

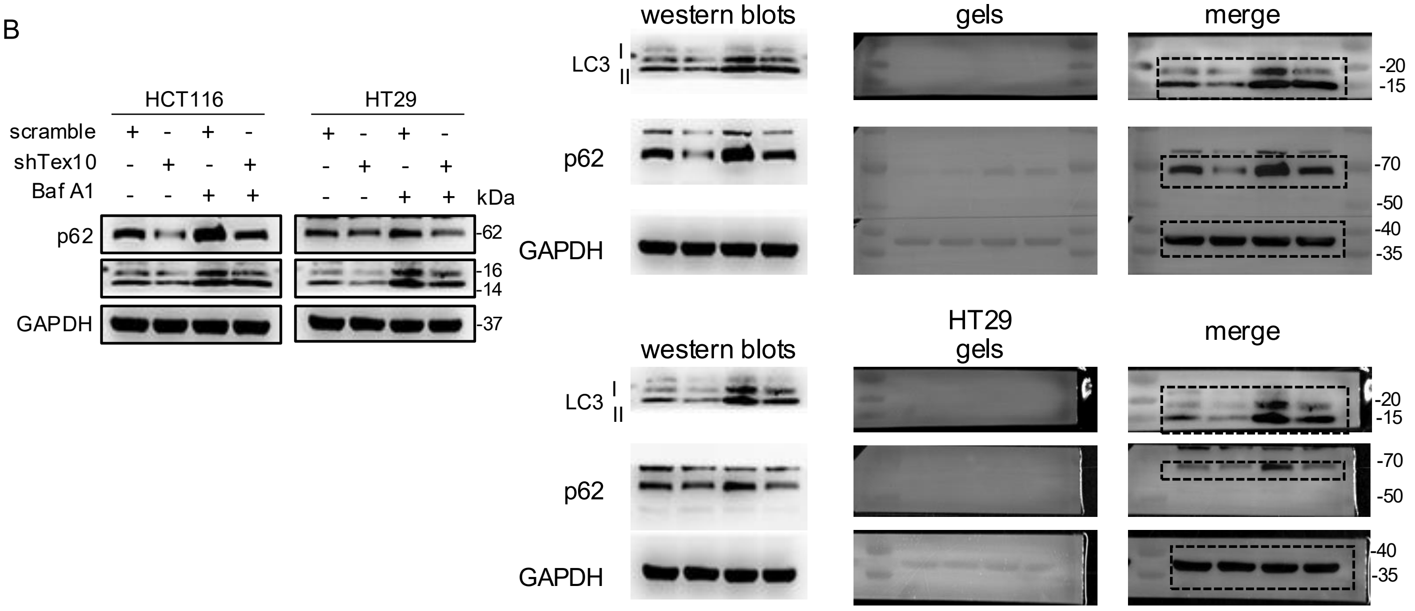

Figure 4A

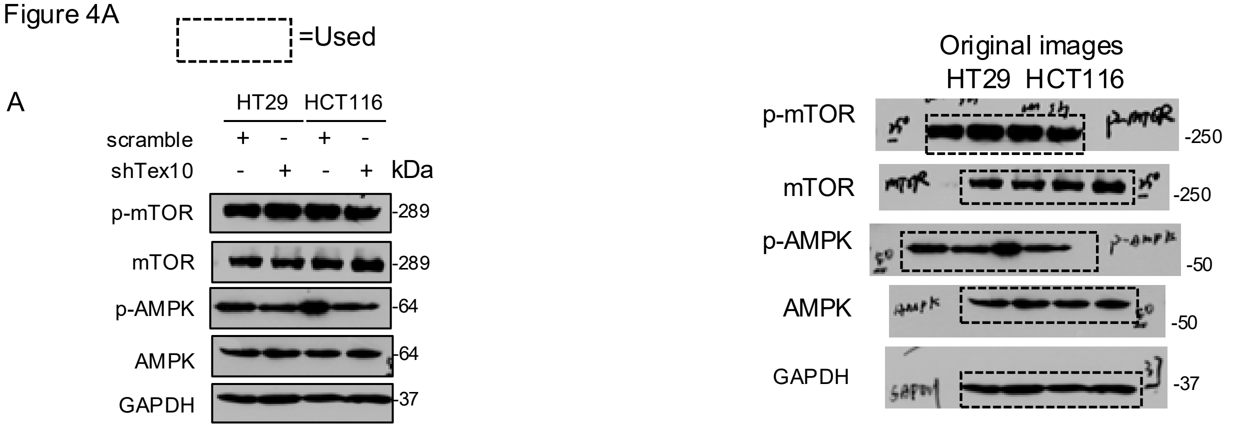

Figure 4C

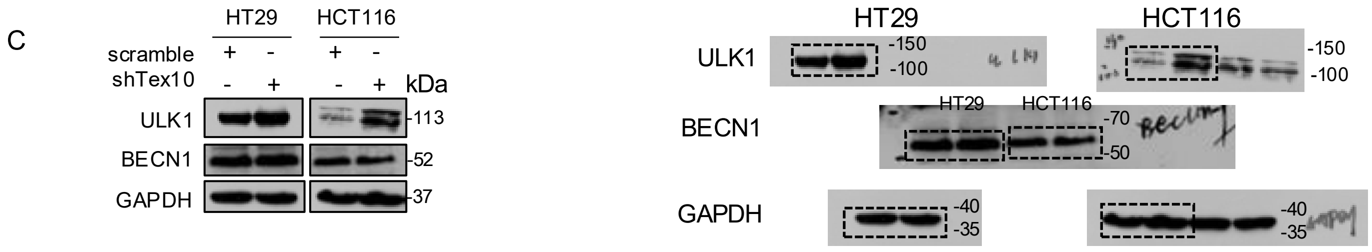

Figure 4D

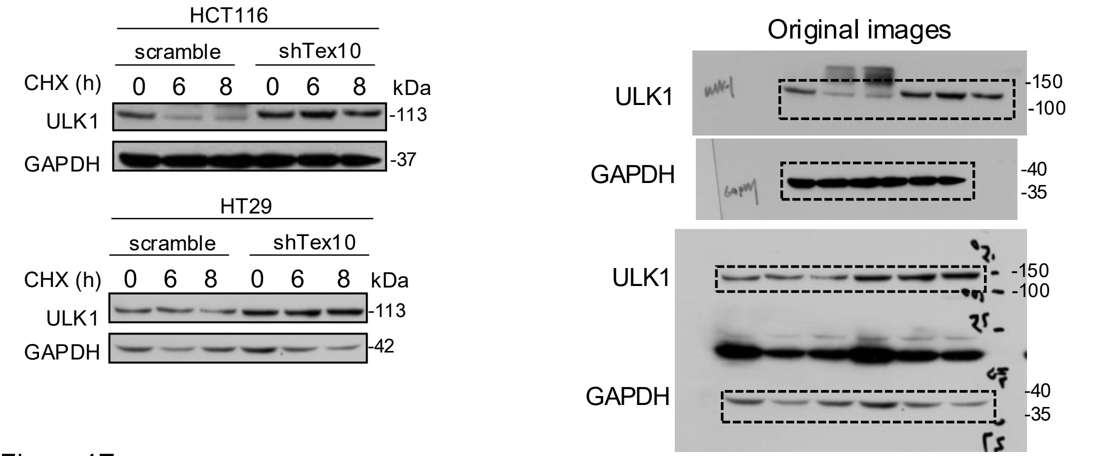

Figure 4E

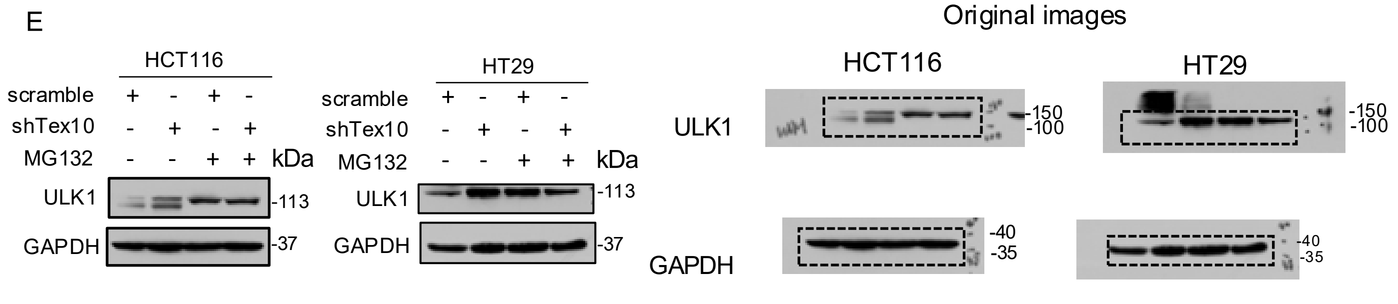

Figure 4F

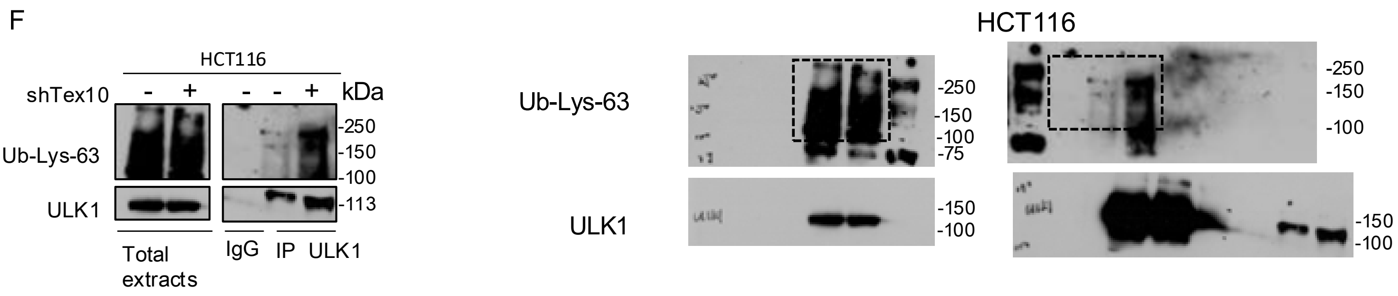

Figure 4G

=Used

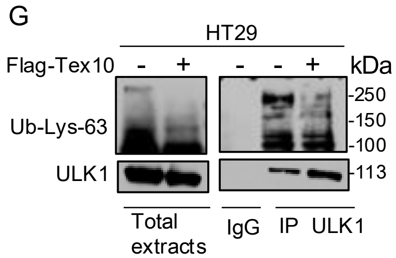

Original images

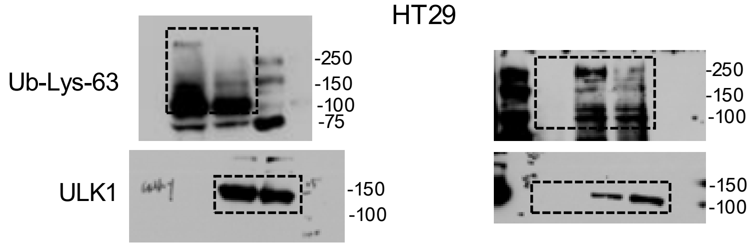

Figure 4J

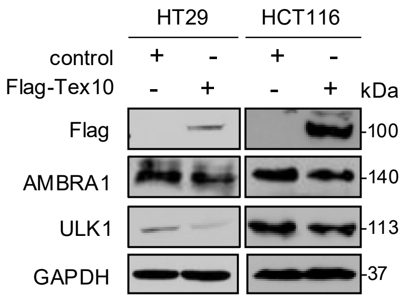

Original images

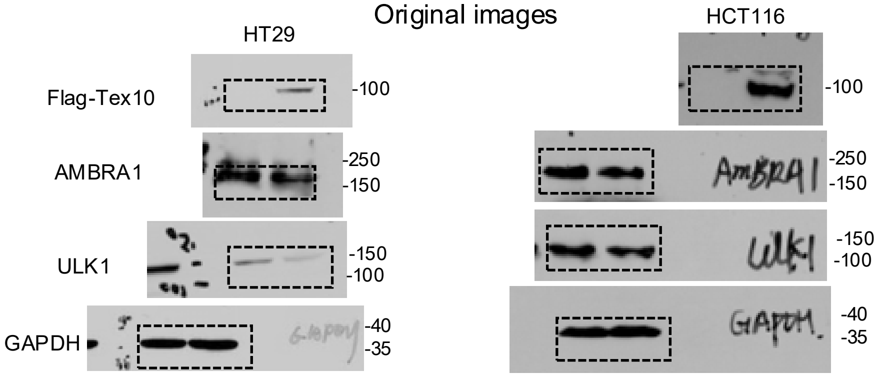

Figure 4K

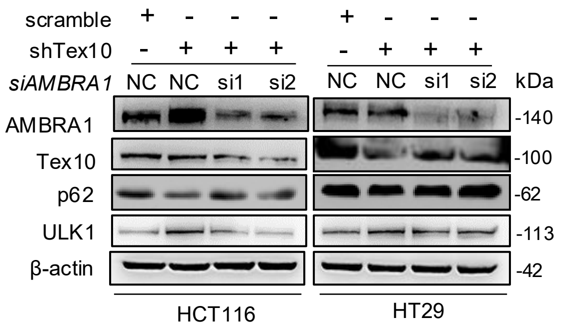

Original images

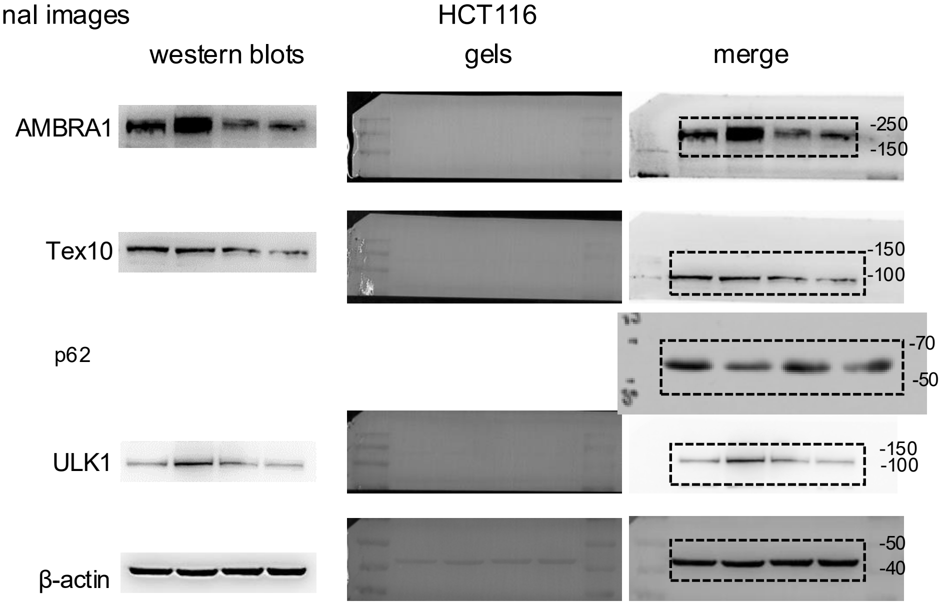

Figure 4K

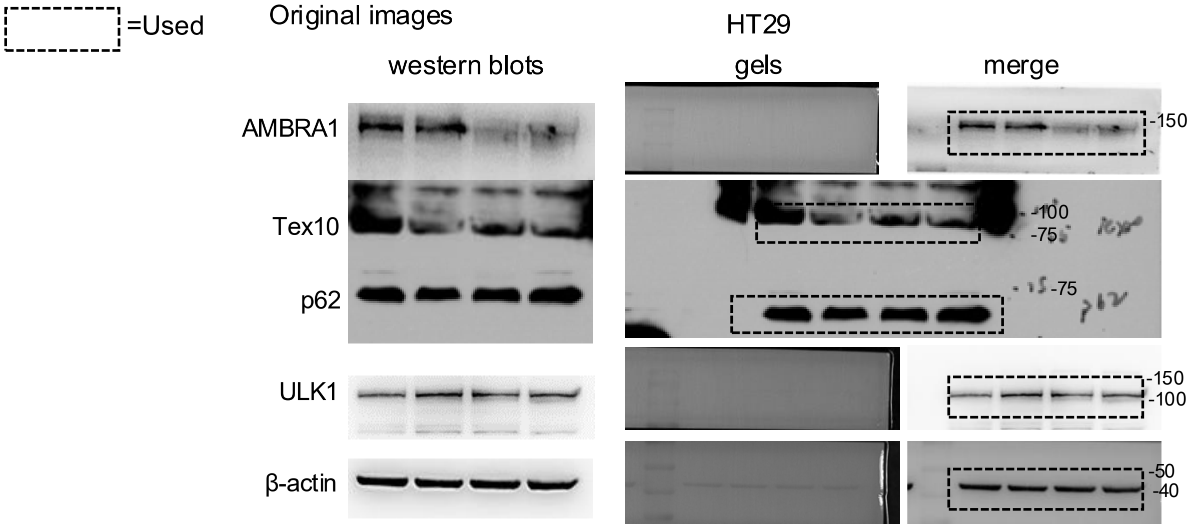

Figure 4M

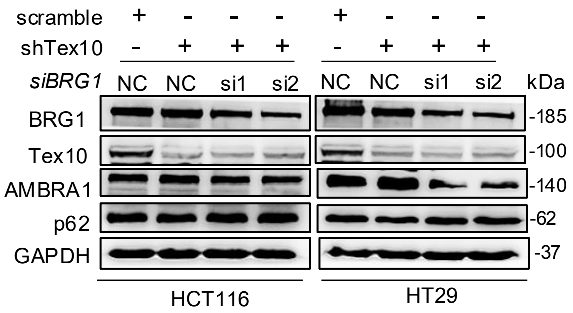

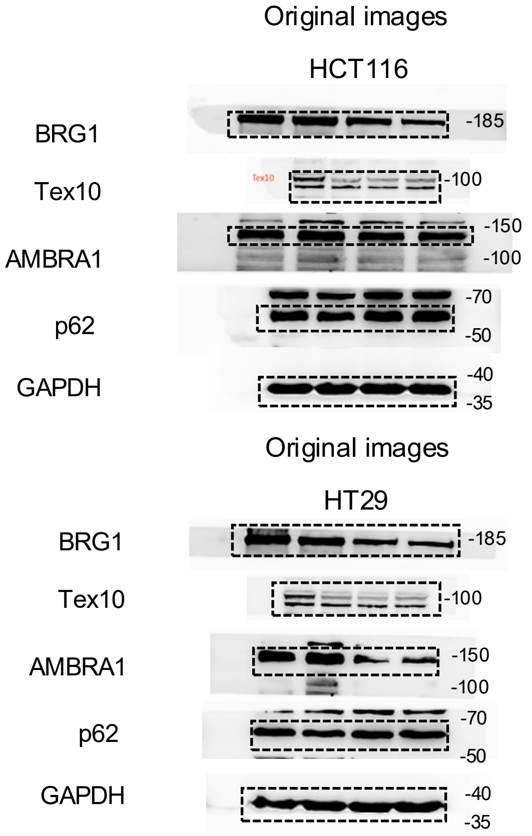

Figure 5B

B

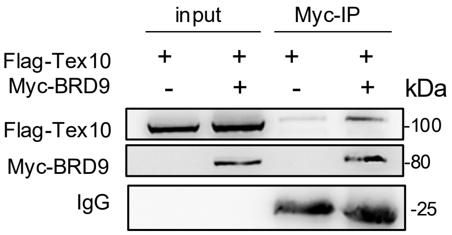

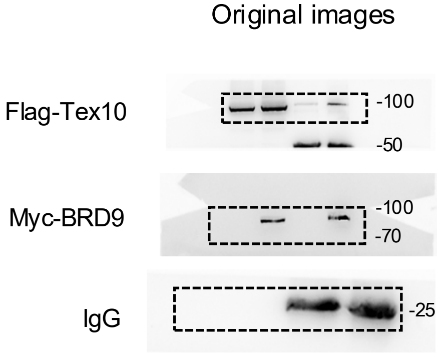

Figure 5C

=Used

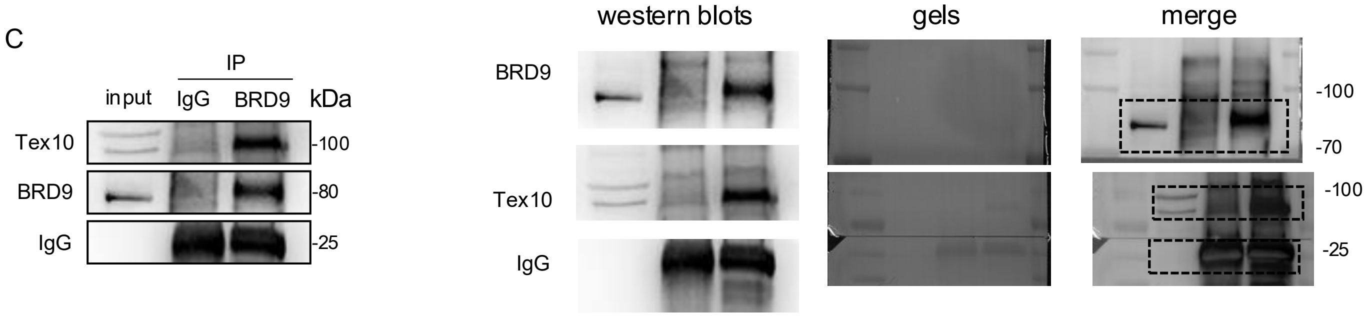

Figure 5E

Original images

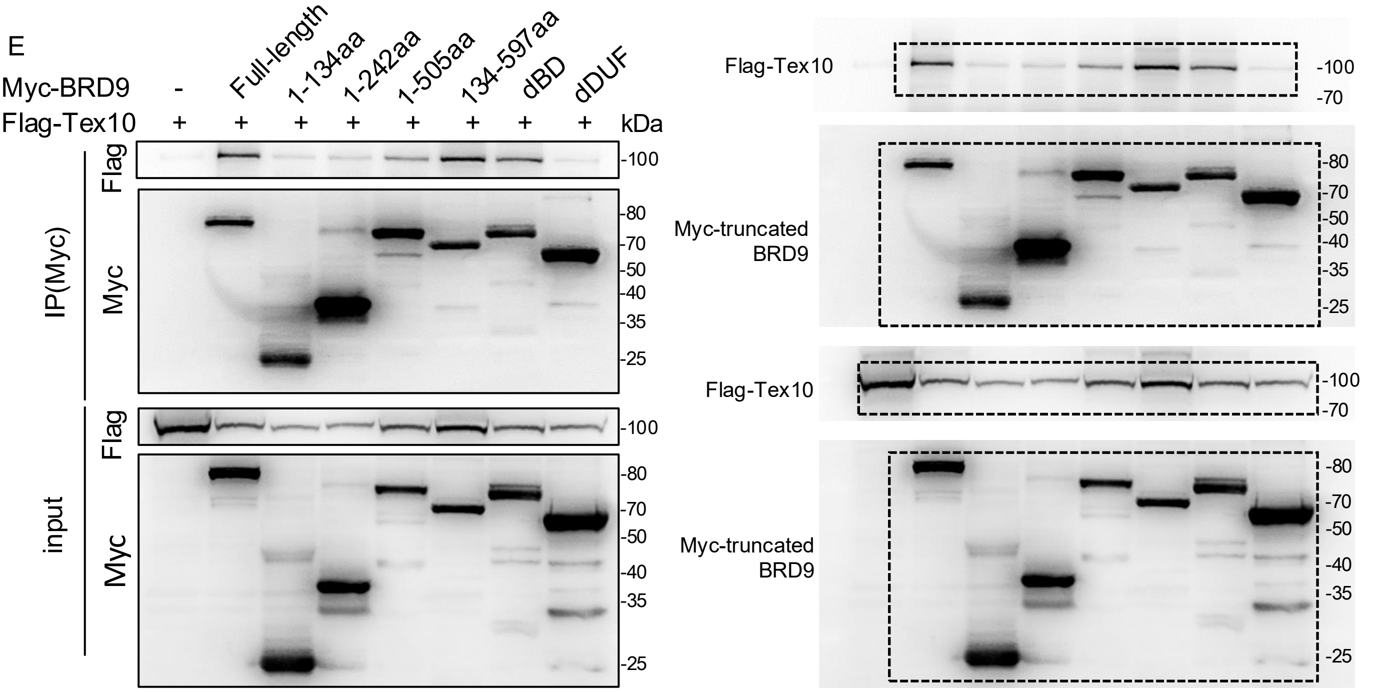

Figure 5F

Original images

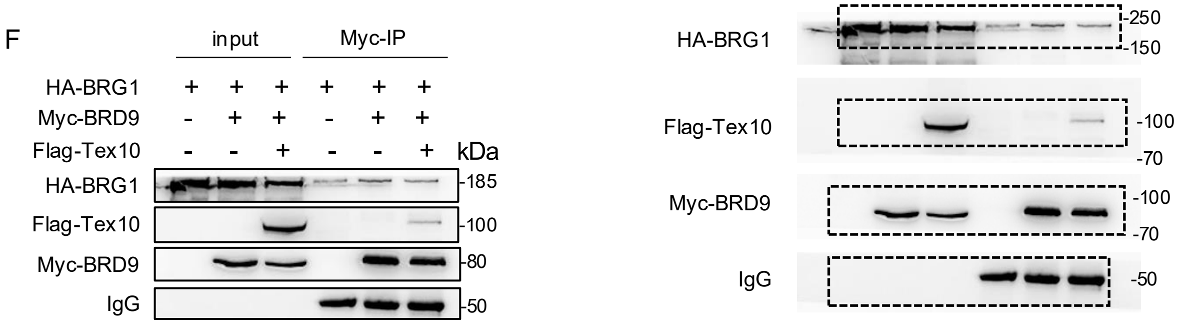

Figure 5H

Original images

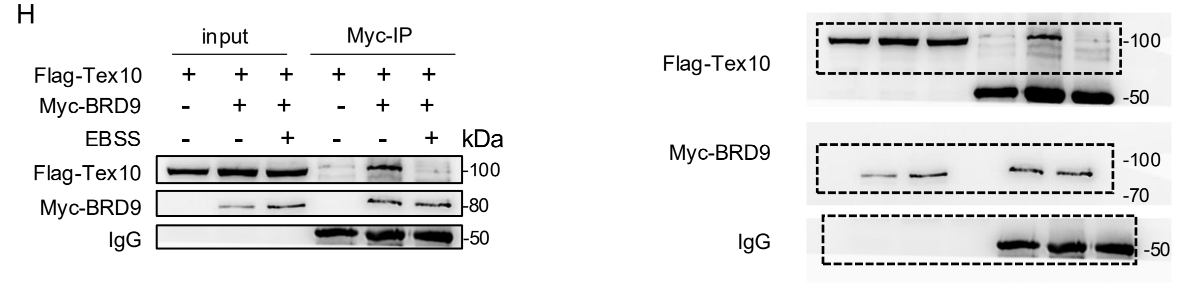

Figure 5G

[dashed box] =Used

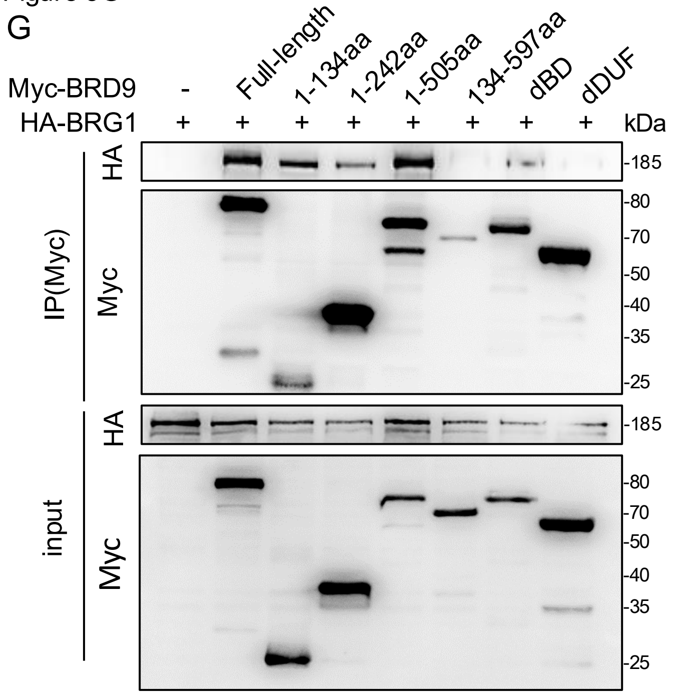

Original images

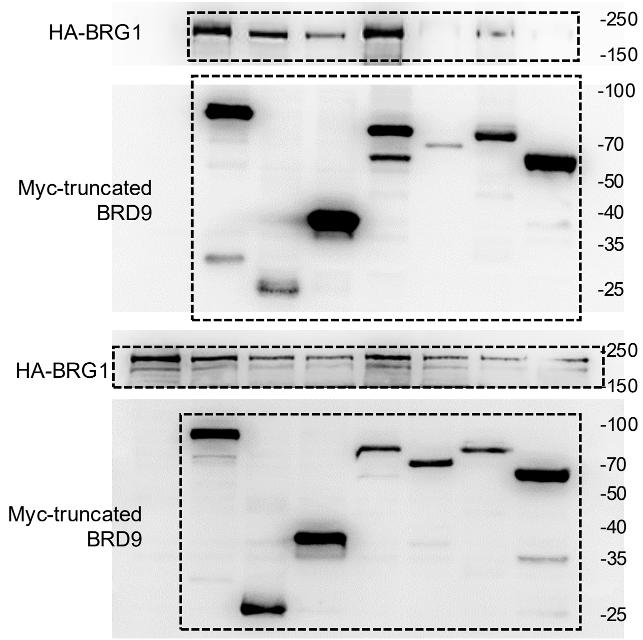

Figure 5I

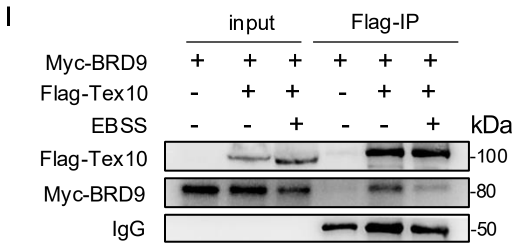

Original images

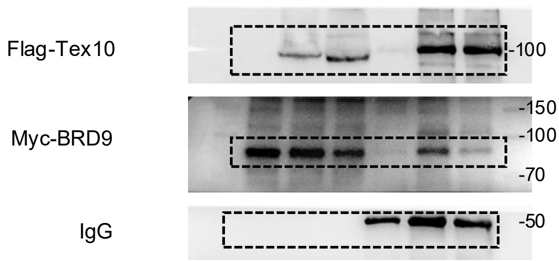

Figure 6B

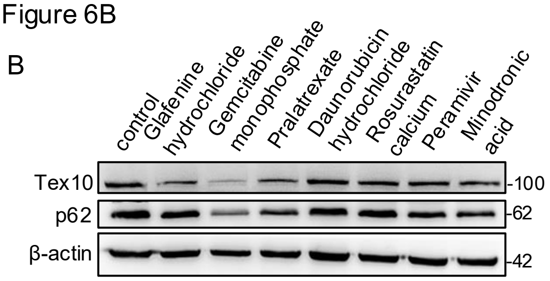

Original images

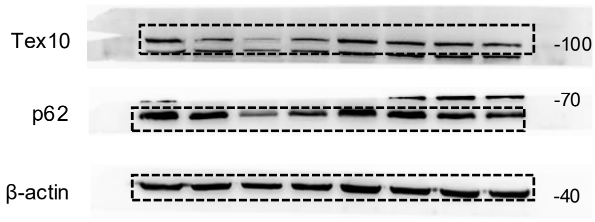

Figure 6D

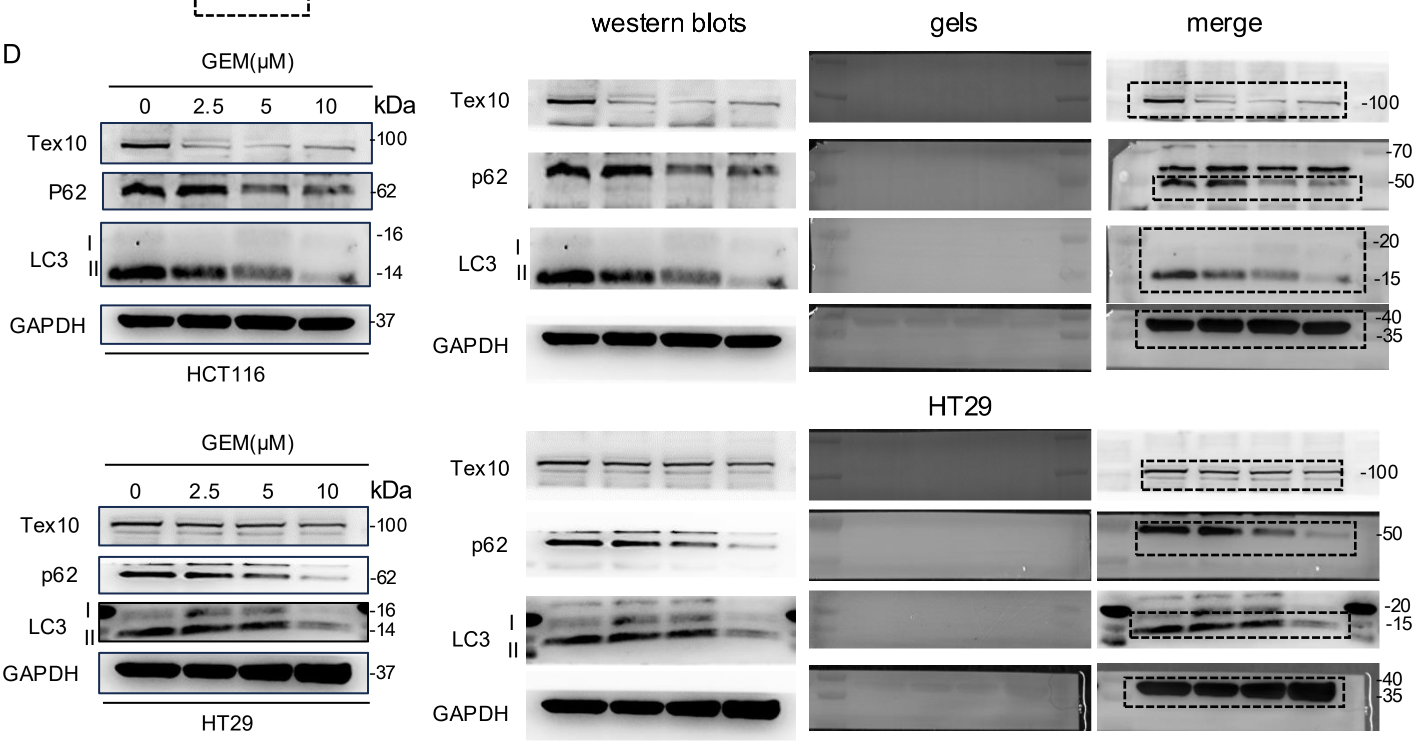

Figure 6E

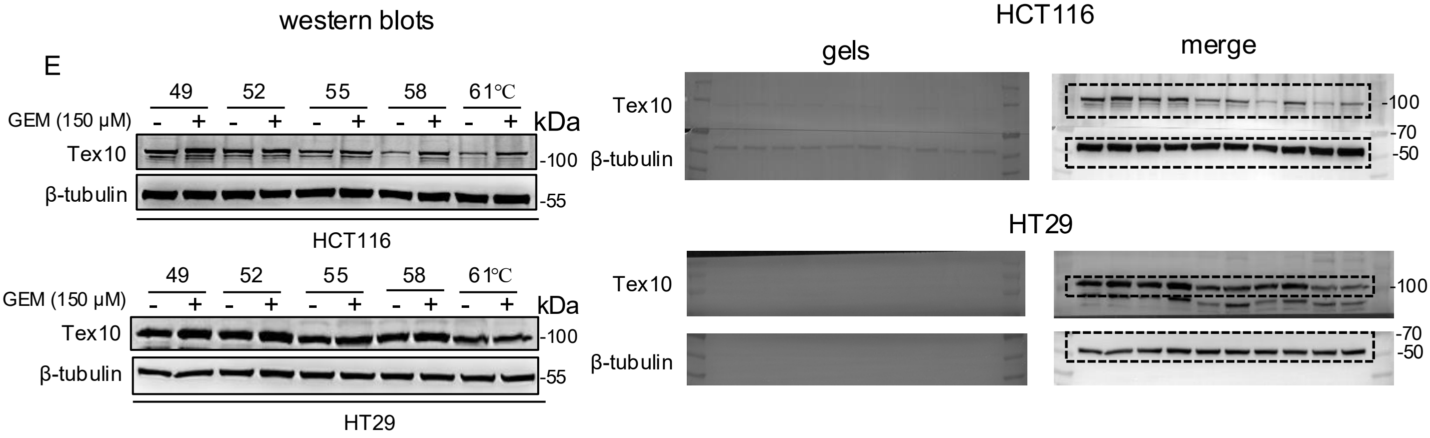

Figure 6F

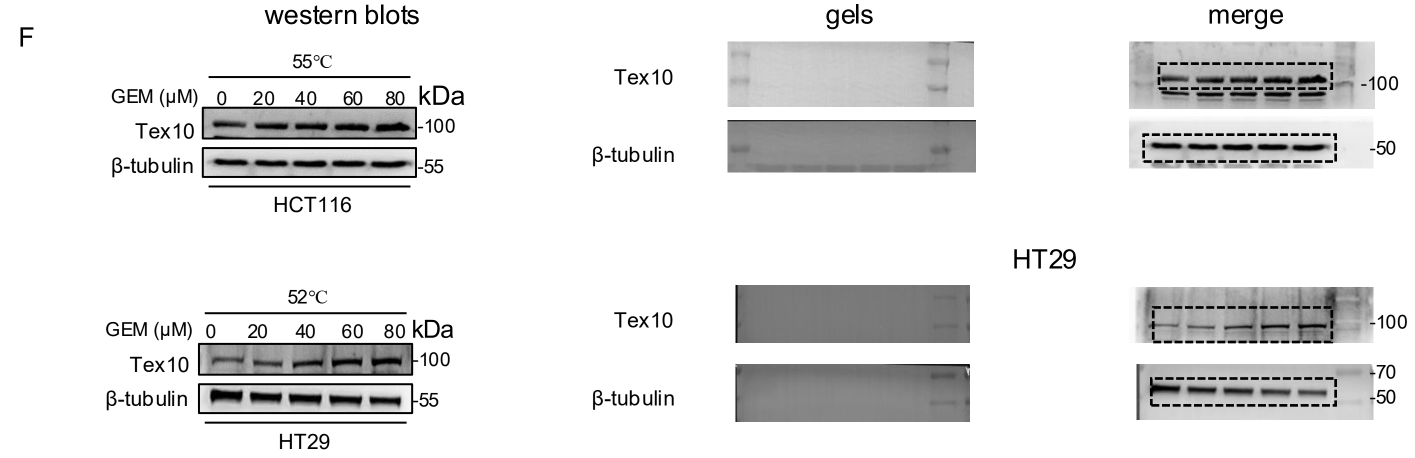

Figure 6K =Used

western blots

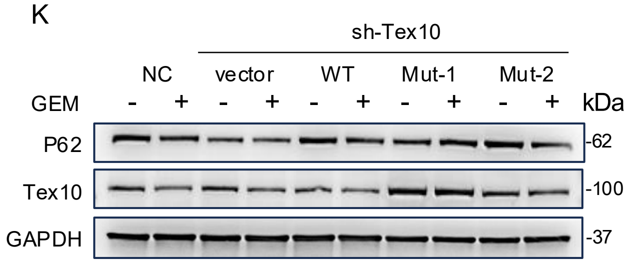

Original images

merge

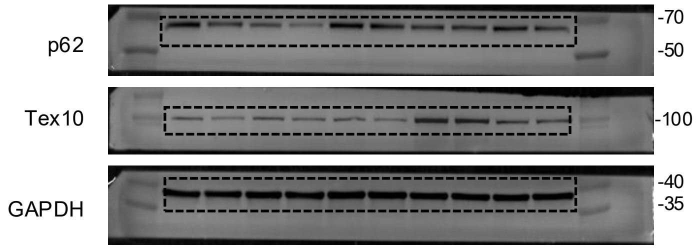

Figure 7B

western blots

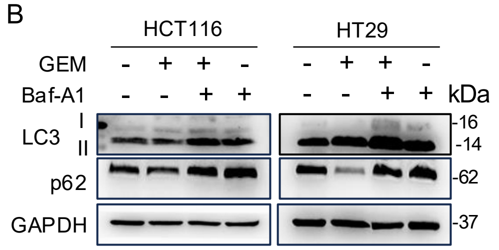

Original images

merge

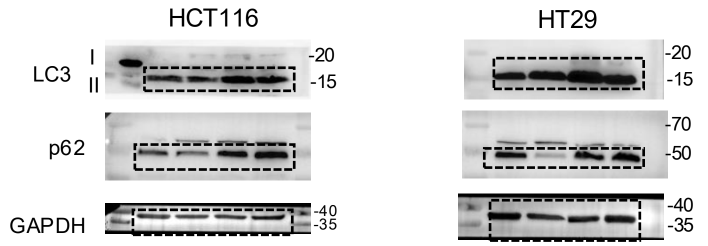

Figure 7C

western blots

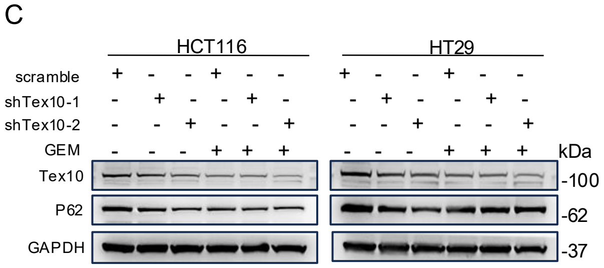

Original images

merge

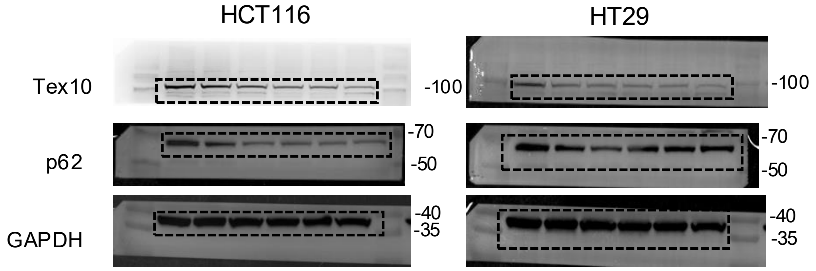

Figure 7D

western blots

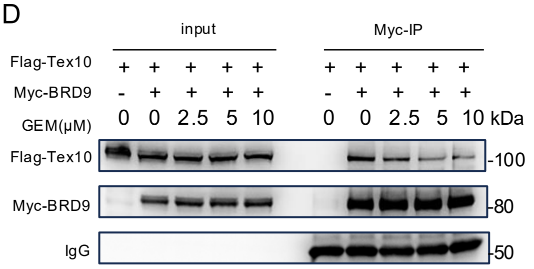

Original images

merge

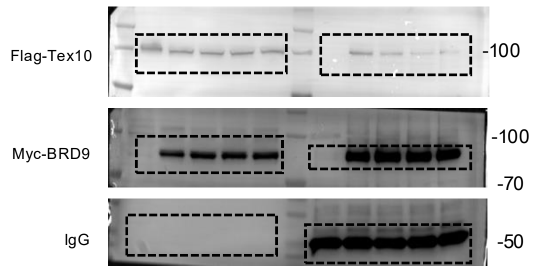

Figure 7F

western blots

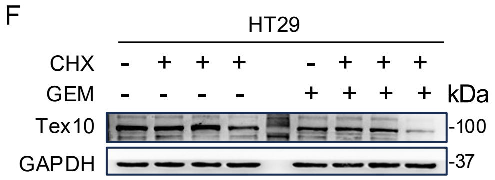

Original images

merge

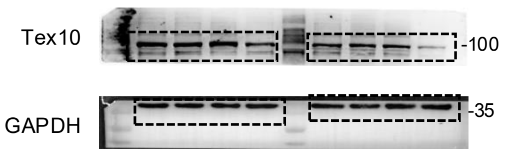

Figure 7G

=Used

Original images

western blots

merge

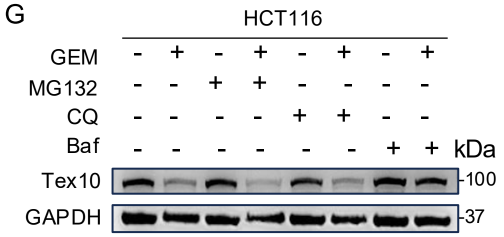

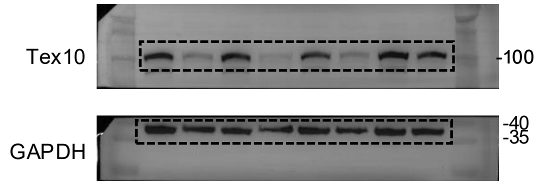

Figure 7H

Original images

western blots

merge

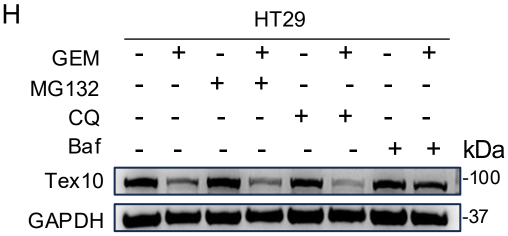

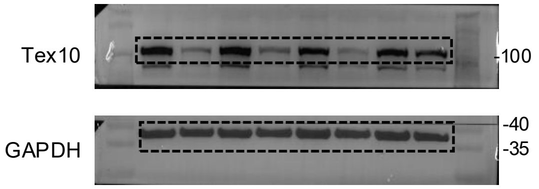

Figure 9A

Original images

western blots

merge

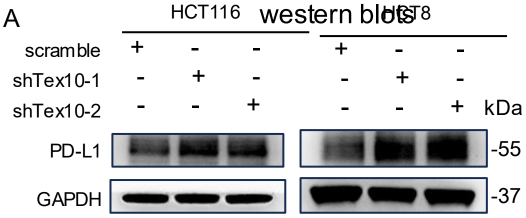

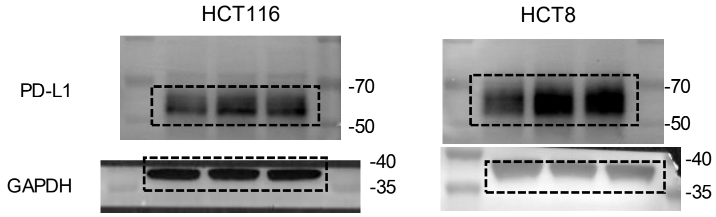

Figure 9C

Original images

western blots

merge

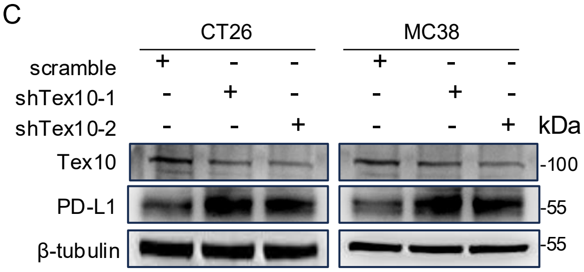

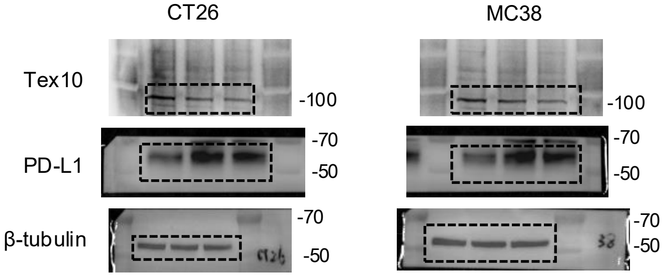

Figure 9E

Original images

western blots

merge

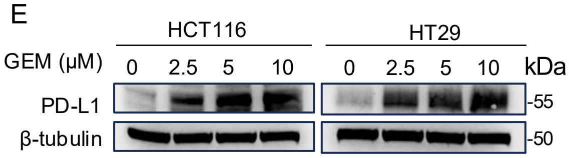

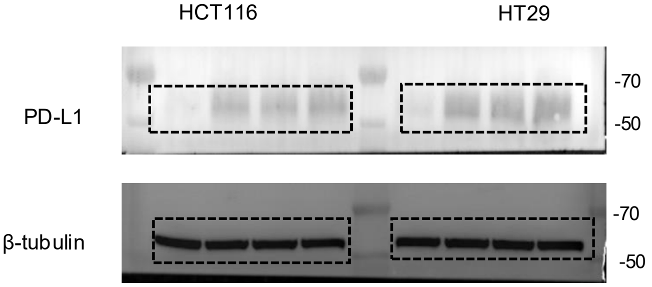

Figure 9K =Used western blots

Original images

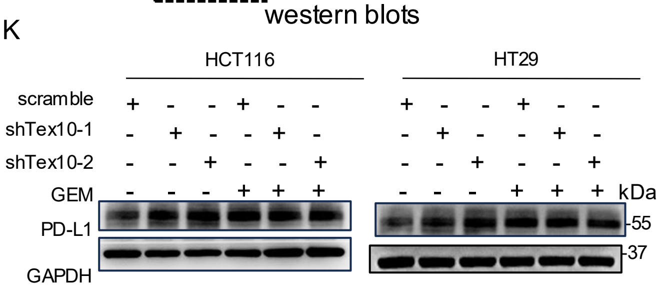

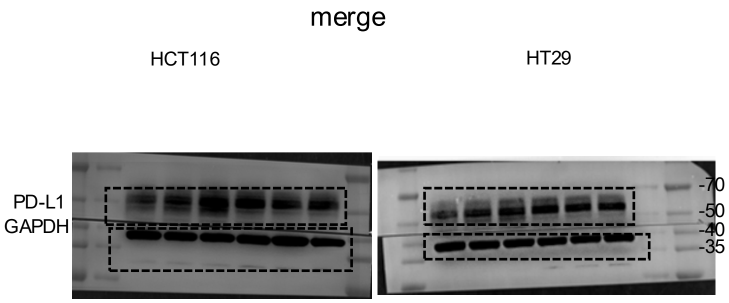

Figure 9I

Original images

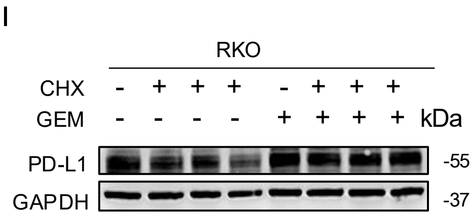

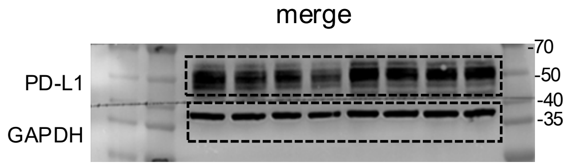

Figure 9J

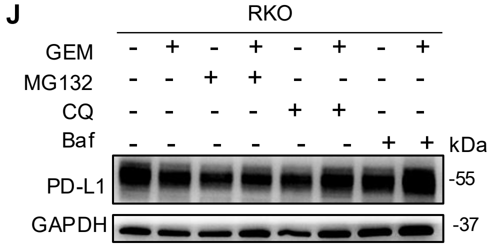

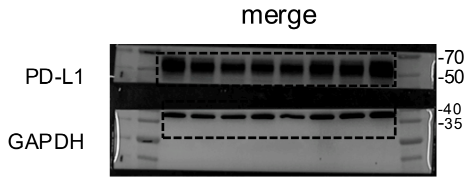

Supplementary Figure

=Used

Figure 2A

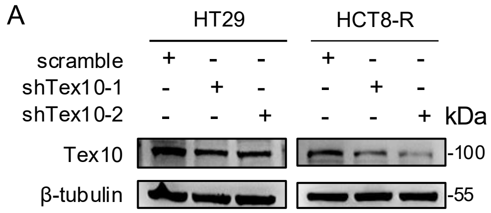

Original images

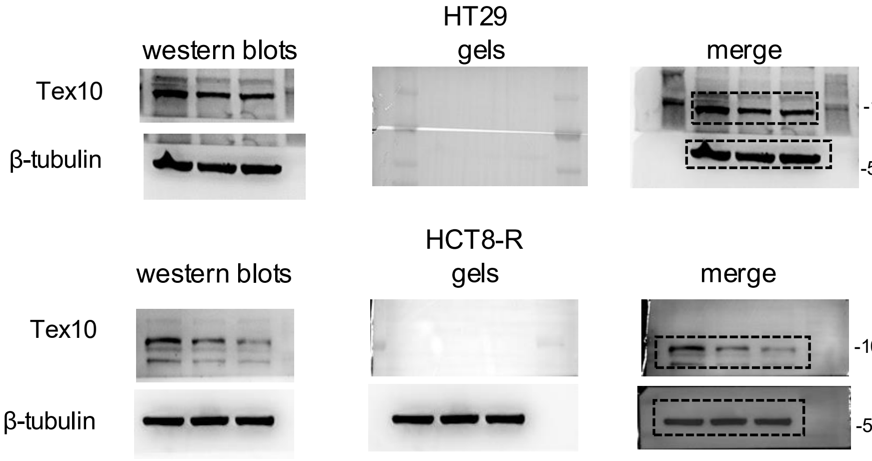

Supplementary Figure

=Used

Figure 3A

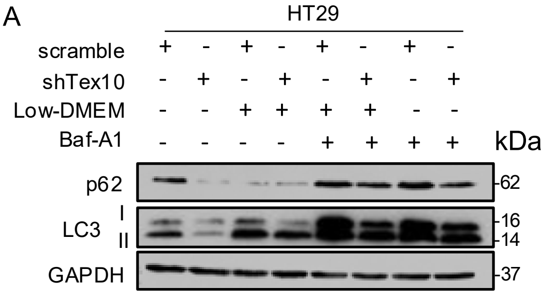

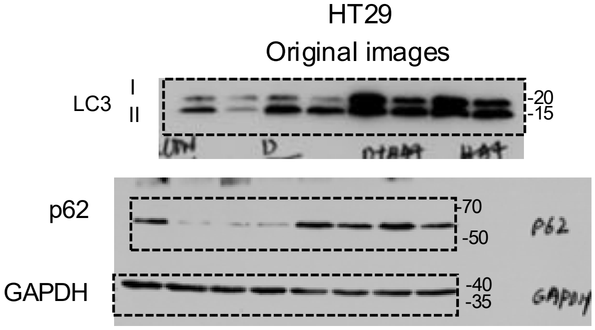

Figure 3B

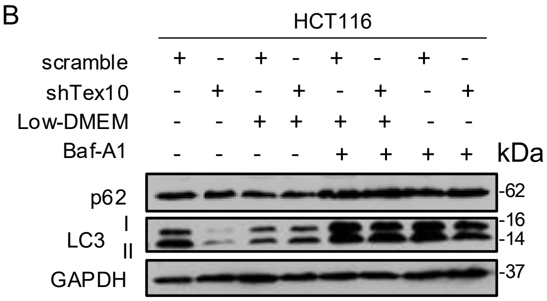

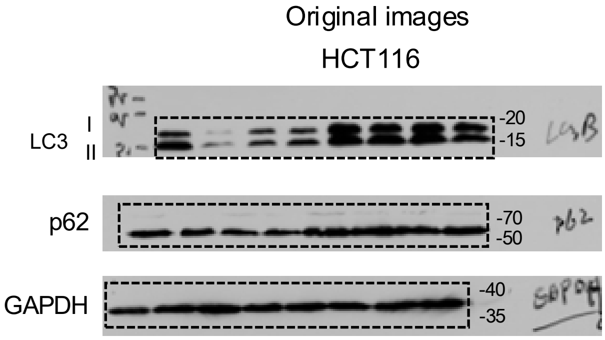

Figure 3C

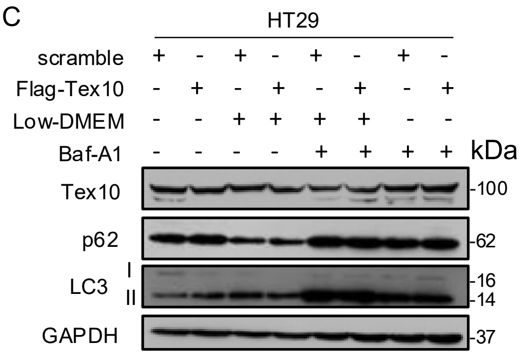

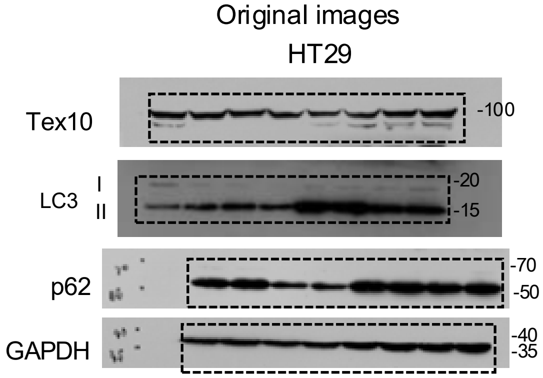

Supplement: Supplementary file 2 — Supporting File 2: advs76895‐sup‐0002‐DataSet.zip. [file ADVS-9999-e76895-s002.zip › Unprocessed western blots-2026-06-1 +.pdf]
